# A unified neural representation model for spatial and conceptual computations

**DOI:** 10.1073/pnas.2413449122

**Published:** 2025-03-10

**Authors:** Tatsuya Haga, Yohei Oseki, Tomoki Fukai

**Affiliations:** ^a^Neural Computation and Brain Coding Unit, Okinawa Institute of Science and Technology, Onna-son, Okinawa 1919-1, Japan; ^b^Center for Information and Neural Networks, National Institute of Information and Communications Technology, Suita-shi, Osaka 565-0871, Japan; ^c^Department of Language and Information Sciences, University of Tokyo, Meguro-ku, Tokyo 153-8902, Japan

**Keywords:** spatial navigation, hippocampus, entorhinal cortex, natural language processing

## Abstract

The hippocampus and entorhinal cortex encode spaces by spatially local and hexagonal grid activity patterns (place cells and grid cells), respectively. In addition, the same brain regions also implicate neural representations for nonspatial, semantic concepts (concept cells). These observations suggest that neurocomputational mechanisms for spatial knowledge and semantic concepts are related in the brain. However, the exact relationship remains to be understood. Here, we show a mathematical correspondence between a value function for goal-directed spatial navigation and an information measure for word embedding models in natural language processing. Based on this relationship, we integrate spatial and semantic computations into a neural representation model called “disentangled successor information” (DSI). DSI generates biologically plausible neural representations: spatial representations like place cells and grid cells, and concept-specific word representations which resemble concept cells. Furthermore, with DSI representations, we can perform inferences of spatial contexts and words by a common computational framework based on simple arithmetic operations. This computation can be biologically interpreted by partial modulations of cell assemblies of nongrid cells and concept cells. Our model offers a theoretical connection of spatial and semantic computations and suggests possible computational roles of hippocampal and entorhinal neural representations.

## Significance

The hippocampus and entorhinal cortex exhibit place-specific and grid-like neural activity patterns (place cells and grid cells) when an animal travels in the physical space. There are neurons that respond to specific nonspatial semantic concepts in the same brain regions, which are called concept cells. How are those neural representations related? In this paper, we propose a unified computational model of spatial navigation and semantic representation learning, demonstrating that the model produces neural representations which resemble place cells, grid cells, and concept cells. Our model suggests a tight theoretical relationship between spatial and semantic neural representations in the brain.

In the brain, place cells in the hippocampus (HPC) and grid cells in the entorhinal cortex (EC) represent spaces by spatially local and hexagonal grid activity patterns, respectively (1–4). Place cells can directly support spatial recognition and memory, whereas grid cells are considered as the basis of robust spatial navigation and path integration. Theoretically, an animal can estimate the direction to a goal when grid representations of a current position and a goal position are given (vector-based spatial navigation) (5). Furthermore, self-position can be estimated by integrating self-motions when sensory information is not available (path integration) (6, 7). These functions are bases of robust spatial navigation by animals. In addition, recent experiments suggest that grid representations in EC appear not only for physical space but also for two-dimensional perceptual or conceptual space [e.g., 2-D visual (8), olfactory (9), social (10) spaces, and pseudowords (11, 12) and objects (13) associated to 2-D structures], and those representations are the basis of vector-based conceptual inference. HPC also exhibits neural representations for conceptual spaces (14, 15).

One of the ultimate goals of the brain is to perform decision making for survival which maximizes rewards (e.g., foods and social success) and minimizes penalty (e.g., hunger and injury), thus various aspects of the brain function (e.g., prediction, abstraction, memory recall, sensorimotor transformation, and behavioral composition) have been understood from the perspective of reinforcement learning theory (16–21). Because spatial navigation is also a special case of decision making, we may hypothesize that such spatial representations in HPC and EC are designed for efficient reinforcement learning. Previous computational studies suggest that successor representation (SR), which was originally proposed for efficient evaluation of value functions (22, 23), can explain various experimental observations of place cells and grid cells (24, 25). Further experiments confirmed the existence of SR-like representations in the brain (25–27). Furthermore, default representations (DR) based on the theory of linear reinforcement learning (28, 29) explain that spatial representations in EC are related to flexible behaviors (30). These theories provide the formal explanation of the design principle of spatial representations like place cells and grid cells, which deepens our understanding of how those representations can cooperate with other brain functions for decision making by animals.

However, in HPC and EC, there are also neurons representing nonspatial semantic concepts which are called “concept cells” (31–33). Concept cells respond to specific concepts, namely, stimuli related to a specific person, a famous place, or a specific category like “foods” and “clothes,” irrespectively of sensory modality (images, written or spoken words). Those cells are activated not only by external stimuli but also by imagery (34) and memory recall (35), suggesting that their activities are not merely sensory responses but conceptual representations of the high-level cognitive process. Because these cells have been found in HPC and EC, it is possible that concept cells can also be understood from the theory of spatial representations, that is, the theory of reinforcement learning. In other words, neural representations of semantic concepts may also be determined by computational principles for decision making. However, how the formal spatial representation model like SR can be extended to such conceptual representations has not been fully understood.

In this paper, we show mathematical correspondence between reinforcement learning and natural language processing (NLP), from which we derive a unified neural representation model called “disentangled successor information” (DSI). We extend SR to a quantity called SI, which is equivalent to both a value function for spatial navigation in linear reinforcement learning (28–30) and an information measure used in word embedding models in NLP (36–39). Therefore, SI can be regarded as an integrated model of those two computational domains. DSI is representation vectors obtained from dimension reduction of SI under biologically plausible constraints such as nonnegativity and decorrelation. In 2-D spaces, DSI forms place and grid representations corresponding to place cells and grid cells, supporting near-optimal decision making for spatial navigation. When we apply DSI to languages, DSI forms word representations in which each unit is activated by a specific concept like concept cells. Therefore, DSI representations can be interpreted as spatial and semantic representations in HPC and EC.

Furthermore, the DSI model offers a common computational mechanism for spatial and semantic inferences. We show that DSI can perform analogical inference of words like word embedding models. Unlike conventional word embedding models, DSI enables the inference by switching only a few units, which can be biologically interpreted as a partial recombination of concept-cell assemblies. Intriguingly, we found that the same computational framework enables analogical inference of spatial contexts by combining previously learned spatial representations. As was suggested in experiments (40), nongrid spatial representations rather than grid representations are crucial for this computation of spatial contexts. Thus, our model provides a shared computational framework behind spatial and semantic inferences as well as a correspondence between nongrid cells and concept cells in biological computation.

Previous computational studies have revealed how spatial (24, 26, 30, 41–46) and conceptual (47–51) representations emerge and function in the brain. However, theoretical understanding of the relationship between spatial and conceptual representations has been scarce, especially for highly complex conceptual structures like languages. Although several models have recently worked on this problem (52–55), it is often difficult to discuss computational properties of neural representations other than place cells and grid cells, such as concept cells (31–33) and nongrid spatial representations that represent contextual information in EC (40, 56). Our model reveals a strong theoretical connection between spatial and semantic computations in the brain, which are biologically interpretable at unit-level and at population-level (*SI Appendix*, Fig. S1). From another perspective, our model suggests that word embedding models in NLP can be grounded on processing in the human brain.

## SI.

Our model is based on the observation that co-occurrence statistics, or prediction of temporally close states, is a shared principle between reinforcement learning and word representation learning. In reinforcement learning, decision making is based on the prediction of future positive and negative rewards from current states and actions (value functions). Intuitively, value functions evaluate the co-occurrence of states and rewards in a certain temporal window. SR evaluates co-occurrence statistics between states, predicting near-future states instead of rewards per se. On the other hand, word representation learning methods in NLP such as Skip-gram and GloVe (36–38) are built on the principle of distributional hypothesis, which argues that words that appear in similar contexts have similar meanings (39, 57). “Context” is defined by the co-occurrence of words: If two words A and B co-occur frequently with the same word repertoire (i.e., the two words equally predict the occurrence of the same words in the near future), they are contextually similar and have similar meanings. Due to this commonality, we may extend co-occurrence-based representations for reinforcement learning (SR) to word representation learning, by which we can obtain a unified view of representations in HPC and EC for spatial navigation and semantic computation. Our model suggests that semantic (word) representations are essentially predictive representations emerging from principles of decision making to represent contextually determined “meanings” of states.

Based on this idea, we introduce a quantity called SI as an extension of SR (22, 24, 25) to directly connect reinforcement learning and semantic learning. Assuming *N_s_* discrete states (positions) exist in the environment, SR between two states *s* and *s*^′^ is defined as

[1] $$\begin{array}{c} SR\left(s,s\prime \right)=E\left[\sum_{t=0}^{\infty }\gamma^{t}\delta \left(s_{t},s^{\prime }\right)|s_{0}=s\right]\\ \;=\sum_{t=0}^{\infty }\gamma^{t}P\left(s_{t}=s^{\prime }|s_{0}=s\right), \end{array}$$

where *δ*(*i,j*) is Kronecker’s delta and *γ* is a discount factor. Based on SR, we define SI and positive SI (PSI) as follows.

[2] $$SI\left(s,s^{\prime }\right)=\text{log}\left(SR\left(s,s^{\prime }\right)\right)-\text{log}\left(P\left(s^{\prime }\right)\right),$$

[3] $$PSI\left(s,s^{\prime }\right)=\text{max}\left\{SI\left(s,s^{\prime }\right),0\right\}.$$

Intuitively, $\log\left(SR\left(s,s^{\prime }\right)\right)$ measures temporal proximity (reachability) between states, and self-information $-\log\left(P\left(s^{\prime }\right)\right)$ normalizes $\log\left(SR\left(s,s^{\prime }\right)\right)$ that tends to increase as a function of the occurrence frequency of the state. PSI neglects weak relationships between distant states by rectifying SI. We determined this mathematical form of SI so that it corresponds to both a value function for goal-directed spatial navigation and an information measure for word embedding models. This indicates that there is a mathematical correspondence between the reinforcement learning and word embedding models.

First, SI corresponds to a value function of linear reinforcement learning (28–30) in a specific setting of spatial navigation. Linear reinforcement learning assumes default policy and imposes additional penalty on deviation from default policy, then we can obtain value functions explicitly by solving linear equations. Let us consider a specific condition in which the environment consists of nonterminal states, and a virtual terminal state is attached to a goal state $s_{G}$ arbitrarily chosen from nonterminal states. When the agent gets to the goal, it transits to the terminal state and obtains positive reward. Furthermore, we assume that rewards at nonterminal states are uniformly negative so that the agent has to take a short path to goal to maximize reward. In this setting for goal-directed spatial navigation, we can obtain value functions $v^{*}\left(s\right)$ in linear reinforcement learning as

[4] $$\lambda^{-1}v^{*}\left(s\right)=\text{log}SR^{d}\left(s,s_{G}\right)-\text{log}P^{d}\left(s_{G}\right)=SI^{d}\left(s,s_{G}\right),$$

where $SR^{d}\left(s,s_{G}\right)$ and $SI^{d}\left(s,s_{G}\right)$ are SR and SI under the default policy, respectively, $P^{d}\left(s_{G}\right)$ is a probability of visiting the state $s_{G}$ under the default policy, and *λ* is a parameter representing the relative weight of penalty on deviation from default policy (see *SI Appendix*, *Extended Methods “Mathematical relationship of SI and reinforcement learning”* for details of derivation). Therefore, SI is proportional to value functions for spatial navigation.

Second, DSI is related to word embedding models in NLP (36–39, 58). In linguistics, pointwise mutual information (PMI) and positive PMI (PPMI) are used to measure the degree of coincidence between two words (39). They are defined as

[5] $$\mathrm{PMI}=\text{log}\left(\frac{P\left(word_{i},word_{j}\right)}{P\left(word_{i}\right)P\left(word_{j}\right)}\right),$$

[6] $$PPMI=\text{max}\left\{PMI,0\right\},$$

where $P\left(word_{i},word_{j}\right)$ is a coincidence probability of two words (in a certain temporal window). It has been proven that dimension reduction of PMI approximates a conventional word embedding model skip-gram (36, 37), and similar performance is obtained using PPMI (39). SI can be written as

[7] $$\begin{array}{c} SI\left(s,s^{\prime }\right)=\text{log}SR\left(s,s^{\prime }\right)-\text{log}P\left(s^{\prime }\right)\\ \;=\text{log}\left(\frac{\sum_{t=1}^{\infty }{\text{γ}}^{t}P\left(s_{t}=s^{\prime },s_{0}=s\right)}{P\left(s\right)P\left(s^{\prime }\right)}\right). \end{array}$$

In this formulation, we can see mathematical correspondence between PMI and SI by regarding words as states ($s=word_{i},s^{\prime }=word_{j}$), thus the correspondence between PPMI and PSI. The difference is how to count coincidence: The coincidence in SI is evaluated with an asymmetric exponential kernel as in SR, in contrast that a symmetric rectangular temporal window is often used in typical word embedding. Because of this relationship, we can expect that representation vectors obtained by dimension reduction of a PSI matrix have similar properties to word embedding models.

## Disentangled SI.

We perform the dimension reduction of the PSI matrix using extensions of nonnegative matrix factorization (NMF) (59) with additional constraints. Then, we finally obtain *D*-dimensional nonnegative vectors $\vec{x}\left(s\right)$ and $\vec{w}\left(s\right)$ (*D* < *N*_s_) which satisfies $\vec{x}\left(s\right)\cdot \vec{w}\left(s^{\prime }\right)\approx PSI\left(s,s^{\prime }\right)$. We call those representation vectors as disentangled SI (DSI). These representation vectors correspond to those obtained in word embedding models because of the relationship between PSI and PMI. Furthermore, from the mathematical correspondence shown in the previous section, $\vec{x}\left(s\right)\cdot \vec{w}\left(s^{\prime }\right)$ approximates a value function of s when $s^{\prime }$ is given as a goal. Therefore, we basically regard $\vec{x}\left(s\right)$ as a representation of each state, and $\vec{w}\left(s^{\prime }\right)$ represents a temporary goal. Below, we call $\vec{x}\left(s\right)$ as a DSI representation vector unless otherwise specified.

In this study, we test two types of constraints for dimension reduction, and we call the resultant vectors as DSI-decorr or DSI-sparse depending on settings. The first one, DSI-decorr was constrained by nonnegativity, decorrelation, and L-2 regularization. Previous theoretical studies have shown that these constraints are important for generation of grid representations (41–46, 60, 61). The other one, DSI-sparse was obtained under nonnegativity and L-1 sparse constraint. A previous study in word embedding suggests that these constraints enable generation of word representation vectors with conceptual specificity (58). Therefore, we can expect that either or both of those constraints achieves simultaneous generation of spatial representations and semantic representations which correspond to grid cells and concept cells, respectively. These constraints are biologically plausible because neural activities are basically nonnegative, and decorrelation and sparsification is possible through lateral inhibition. For details of dimension reduction, see *SI Appendix*, *Extended Methods “Details of dimension reduction for DSI vectors.”*

## Emergence of Spatial Representations Like Grid Cells and Place Cells.

**Fig. 1.**
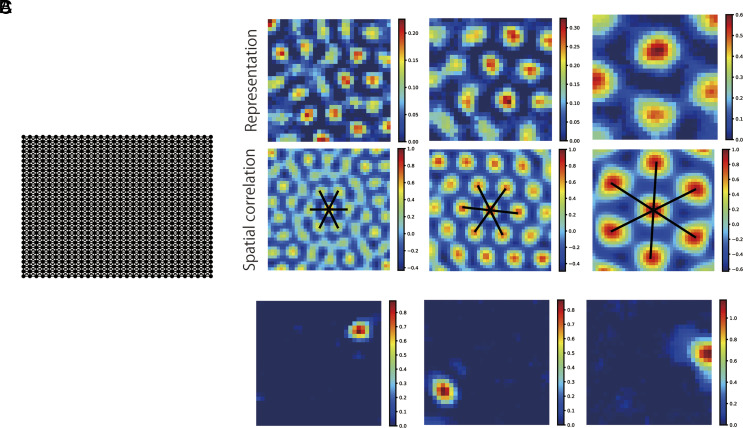
DSI representations for the space. (*A*) The square room tiled with 30 × 30 discrete states. (*B*) Grid-like spatial representations generated by DSI-decorr (*Upper*) and their spatial autocorrelation (*Lower*). (*C*) Spatial representations like place cells generated by DSI-sparse.

Below, we demonstrate properties of DSI representations through simulations. First, we checked spatial representations that the DSI model forms in a 2-D space. As an environment, we assumed a square room tiled with 30 × 30 discrete states (Fig. 1*A*). We generated sequences of states by random walks in the room, from which we calculated 100-dimensional DSI representation vectors for states (places) (see *SI Appendix*, *Extended Methods “Learning DSI in 2-D spaces”* for the detail of the simulation).

Here, we call each dimension of DSI representation vectors $\vec{x}\left(s\right)$ as a neural “unit,” and we regard a value in each dimension at each state as a neural activity (or a neural representation). Then, in DSI-decorr vectors, many units exhibited grid activity patterns in the space (Fig. 1*B*). We performed a gridness analysis that has been used in previous studies (43, 62–64) and found that 27.6 ± 5.6% of units (average ± std of 5 simulations with different random seeds) were classified as grid cells. Similarly, 30.6 ± 4.0% of units in *w*(*s*) were classified as grid cells. We observed similar ratios of grid cells (20 to 30%) in various simulation settings (discount rate, dimensionality, and simulation lengths for learning) although small discount rate γ (γ≤0.98) impaired the emergence of grid cells (*SI Appendix*, Fig. S2). This was presumably because a small discount rate is disadvantageous for sensing the global spatial structure of the environment. On the other hand, DSI-sparse generated spatially local representations which resemble hippocampal place cells (Fig. 1*D*). Therefore, we regard DSI-decorr and DSI-sparse as models of EC and HPC representations, respectively. Below, we mainly show results of DSI-decorr but DSI-sparse also gave qualitatively similar performances in most cases.

## Path Integration and Spatial Navigation by DSI Representations.

**Fig. 2.**
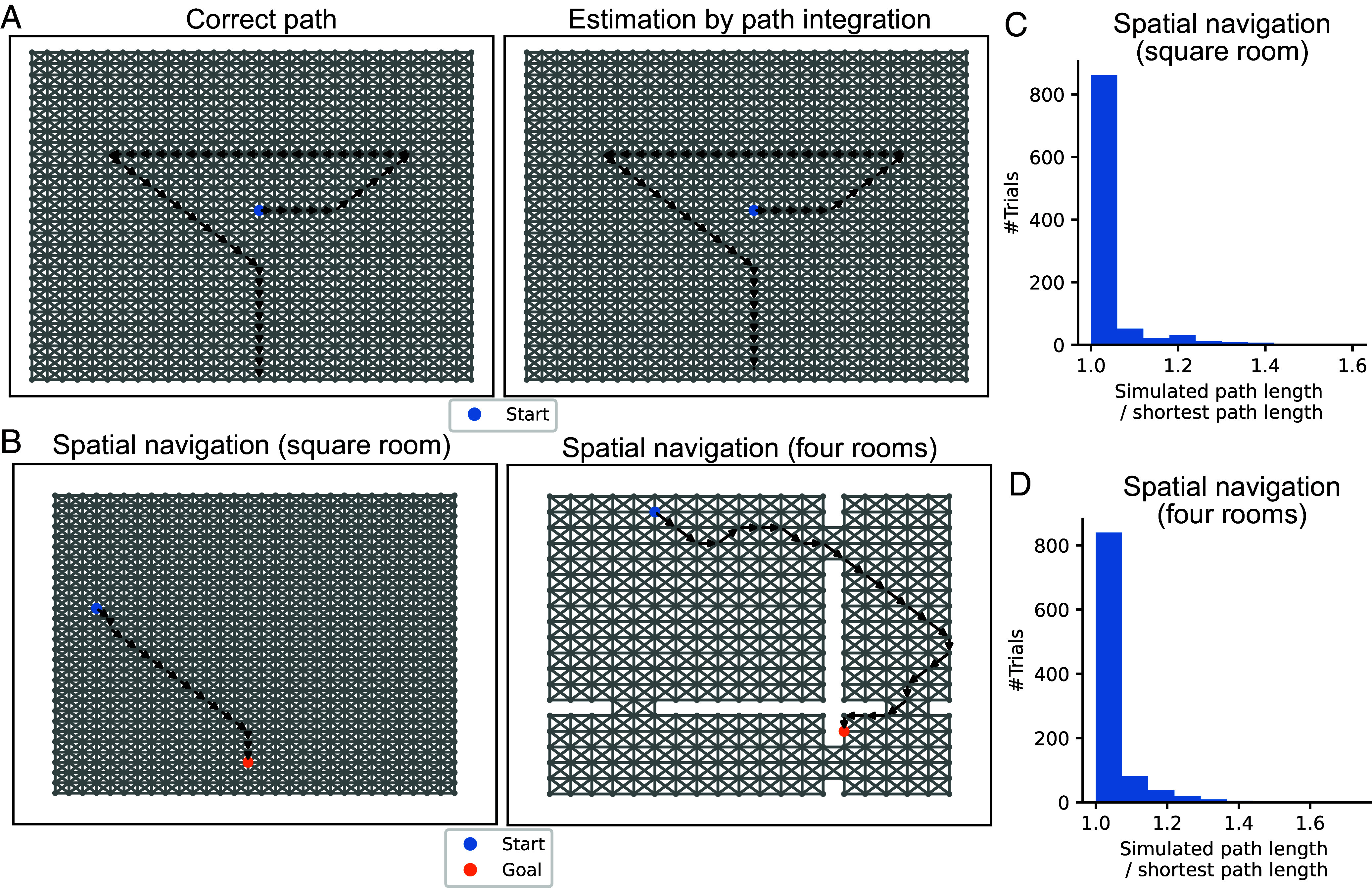
Spatial navigation using DSI representation vectors (DSI-decorr). (*A*) Path integration using the DSI model. (*Left*) Actual path. (*Right*) Path estimated from DSI vectors updated by movement information. (*B*) Example spatial paths obtained by DSI-based navigation. (*C*) A histogram of path lengths in 1,000 trials of spatial navigation in the square-room environment. Note that a start and a goal were randomly determined in each trial, and we normalized a simulated path length by the shortest path length between the start and the goal. (*D*) A histogram of path lengths in the four-room environment.

If DSI representations are a plausible model of place cells and grid cells, they should support path integration and spatial navigation, which are important functions of HPC and EC. First, we performed path integration using DSI representations. In the path integration task, after a starting location (state) is given, an agent has to estimate the current position of itself by integrating only self-movement information. To solve the task, we estimated spatial representations by movement-conditional recurrent weights at each time step (see *SI Appendix*, *Extended Methods “Path integration by DSI vectors”* for details). This strategy has been used in previous studies such as grid cell modeling (45) and action-conditional video prediction (65). This mechanism is also consistent with a conventional biological model for path integration in which head direction signals activate one of attractor networks specialized for different directional shifts of grid patterns (6, 7, 42). As shown in Fig. 2*A* (DSI-decorr) and *SI Appendix*, Fig. S3 (DSI-sparse), this strategy gave accurate estimation of the spatial path from movement signals. To quantify the accuracy, we evaluated the success rate of estimation of spatial paths generated from random starting points and sequences of 10 random movements. Estimation was correct in 969.4 ± 8.3 trials (DSI-decorr) and 759.2 ± 14.4 trials (DSI-sparse) out of 1,000 trials (average ± std of 5 simulations with different random seeds), which suggests that path integration was highly accurate especially with DSI-decorr.

Next, we tested spatial navigation by DSI representations. We assume that a start location and a goal location are randomly given in each trial and an agent has to navigate between them. To solve the task, we defined a vector-based state transition rule that approximates value-based decision making based on the relationship between DSI and value functions (see *SI Appendix*, *Extended Methods “Goal-directed spatial navigation by DSI vectors”* for details). Because of the constraints in the model and the approximation error, this rule did not always give optimal navigation (the shortest path from the start to the goal). However, the agent could take relatively short paths (Fig. 2 *B*, *Left* and *SI Appendix*, Fig. S3) and simulated path lengths were close to the shortest path between the start and the goal (Fig. 2*C* and *SI Appendix*, Fig. S4). Furthermore, the agent could also perform near-optimal spatial navigation in a structure with separated and interconnected rooms (Fig. 2 *B*, *Right*, Fig. 2*D*, and *SI Appendix*, Fig. S3). The agent could also appropriately navigate complex mazes (*SI Appendix*, Fig. S15). These results suggest that DSI representations can support efficient spatial navigation.

We additionally evaluated the performance of path integration and spatial navigation by representation vectors obtained in different parameter settings (discount rate, dimensionality, and simulation lengths for learning). The performance did not largely differ across settings except that small discount rate γ (γ≤0.98) impaired the performance of both tasks (*SI Appendix*, Fig. S2), similarly to failure in generating grid cells. A large value of γ likely makes it easy to sense a long-range spatial structure necessary for effective path integration.

## Emergence of Concept-Specific Representations for Words.

Next, we show that the same DSI model can learn conceptual representations from linguistic inputs. We used text data taken from English Wikipedia, which contains 124M tokens and 9,376 words (see *SI Appendix*, *Extended Methods “Learning DSI from text data”* for the detail of preprocessing). To construct DSI representations, we regarded each word as a state, and considered the text data as a sequence of 9,376 states (*N_s_*= 9,376). Then, we applied the same learning procedure as in the experiment of 2-D spaces. We obtained 300-dimensional DSI representation vectors for each word.

**Fig. 3.**
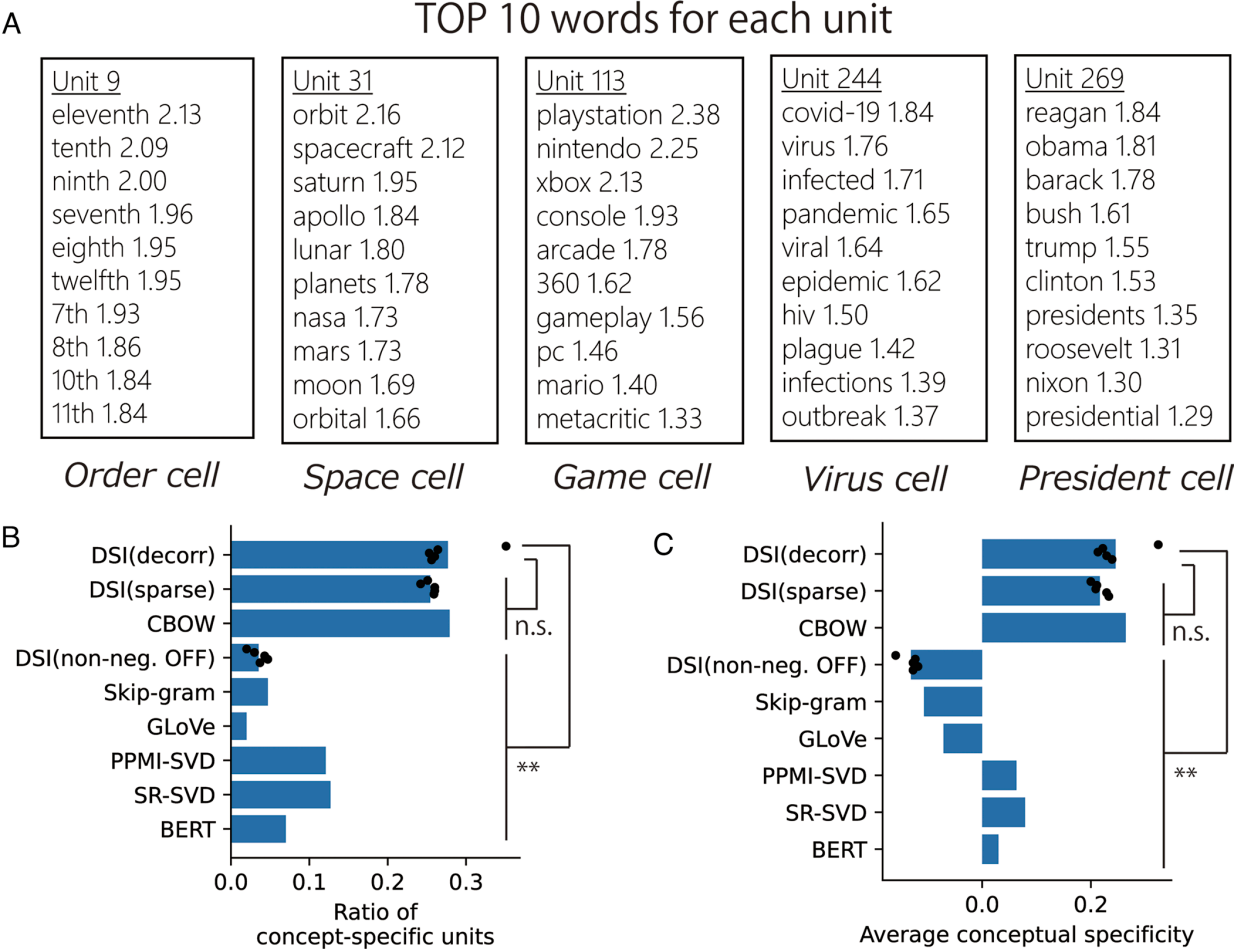
Concept-specific representations formed by DSI-decorr. (*A*) Ten words that gave the highest activation (TOP-10 words) are shown. We also marked each unit with a descriptive label. (*B*) Ratio of concept-specific units (dimensions) in word representation vectors obtained by various methods. For DSI, dots indicate 5 trials with different random seeds (different initial values for learning); bars indicate means of those 5 simulations. We compared DSI-decorr and other DSI models by 2-sample *t* tests, and DSI-decorr and other word embedding methods by 1-sample *t* tests. (*C*) Average conceptual specificity of all units (dimensions) in word representation vectors obtained by various methods. We performed the same statistical analyses with (*B*). **P < 0.01; n.s., not significant. All statistical tests were two-sided *t* tests and significance thresholds were modified by Bonferroni correction. Details of statistical analyses are shown in *SI Appendix*, Table S1.

As in the case of spatial representations, we regard each dimension of representation vectors as a neural unit, and checked how various words activate those units. Specifically, we listed 10 words that elicited the highest activities in each unit (TOP-10 words). Then, we found that many units are activated by words related to specific concepts (Fig. 3*A*; other examples in *SI Appendix*, Fig. S5), which could be named as “game cell” or “president cell,” for example. We quantified this conceptual specificity through WordNet-based semantic similarity between words (66). We compared mean similarity among TOP-10 words and a null distribution of similarity between random word pairs, by which we determined statistically significant concept-specific units and quantified the degree of conceptual specificity of each unit (see *SI Appendix*, *Extended Methods “Quantitative evaluation of conceptual specificity”* for details). As shown in Fig. 3 *B* and *C*, both DSI-decorr and DSI-sparse exhibited the larger number of concept-specific units and higher average conceptual specificity than other well-established word embedding models such as skip-gram and GloVe (36–39). We also analyzed conceptual specificity of representations in the embedding layer of pretrained Bidirectional Encoder Representations from Transformers (BERT) model (67, 68), which was lower than DSI. This result shows that the DSI model forms more concept-specific representations than typical word embedding models in NLP. Remarkably, removal of the nonnegativity constraint from DSI-decorr significantly decreased conceptual specificity. This result suggests that nonnegativity is an essential constraint for concept-specific word representations as well as hexagonal grid spatial representations (41, 42, 60), and sparsity is not necessary as proposed in the previous study (58). It is also notable that continuous bag-of-words (CBOW) model (37) showed almost the same conceptual specificity with DSI. However, we see a functional difference between concept-specific representations in DSI and CBOW later.

We additionally performed the same analyses on word representations with different dimensionality (*D* = 100, 200) and confirmed that conceptual specificity of DSI was higher than other methods (*SI Appendix*, Figs. S6 and S7). Conceptual specificity of CBOW was lower than DSI in other settings, showing that conceptual specificity of CBOW is not robust against the change of settings. We could also obtain concept-specific representations using a different text dataset [WikiText-103 dataset (69)] in which the number of tokens (87M tokens) and the number of embedded words (7,517 words) were different from the main dataset we used (*SI Appendix*, Fig. S8). We also confirmed that changing γ from 0.9 to 0.8 did not change the qualitative results, however, too large γ (γ≥0.95) impaired the emergence of concept-specific representations (*SI Appendix*, Fig. S8), presumably due to difficulty in precise evaluation of word co-occurrence. Thus, the performance depends on the setting of the discount rate γ.

## DSI Representation Vectors Capture the Semantic Structure of Words.

**Fig. 4.**
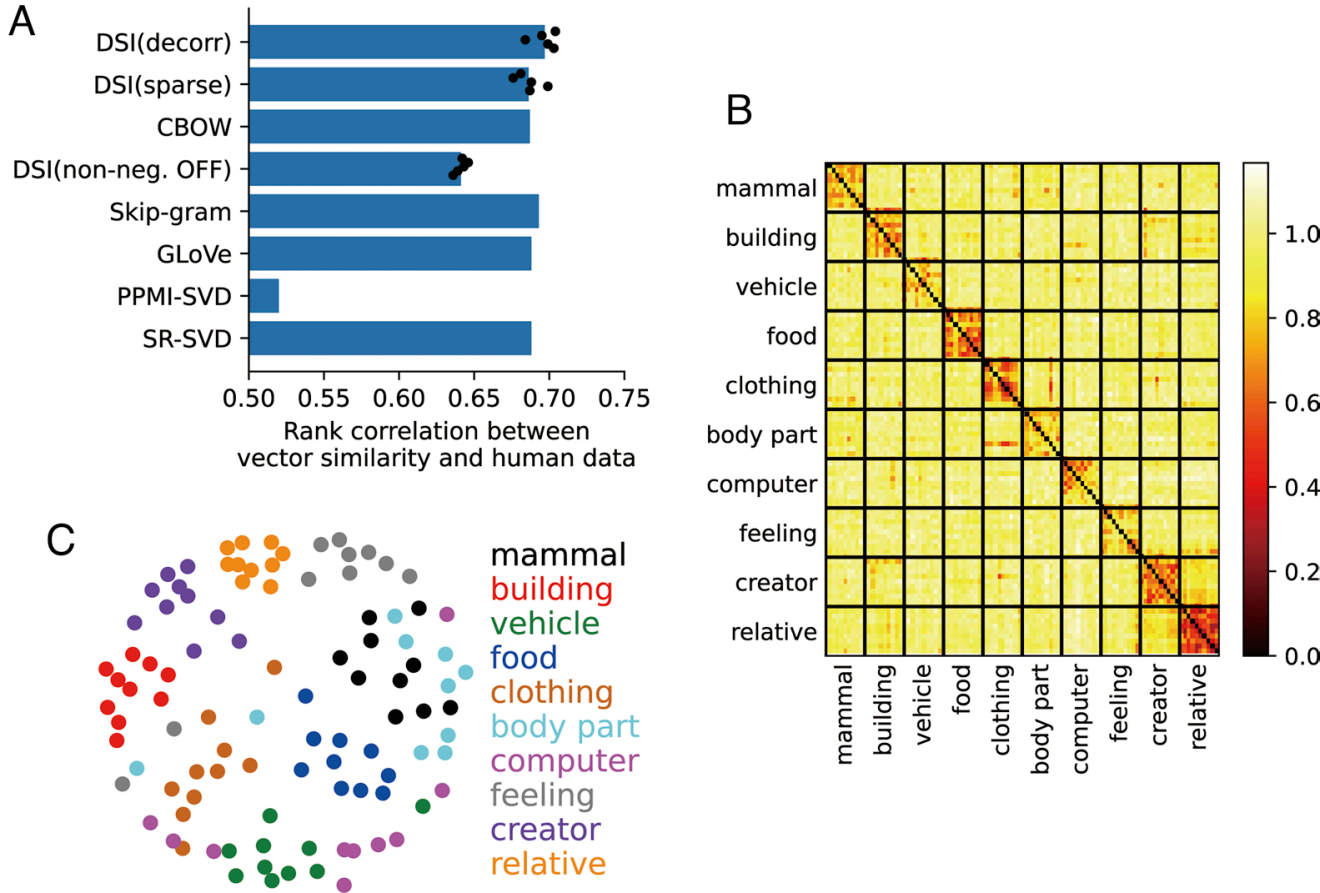
DSI representations capture the semantic structure of words at the population level (DSI-decorr). (*A*) The rank correlation of word similarity evaluated by word representation vectors (cosine similarity) and humans (WS353 dataset). For DSI, dots indicate 5 trials with different random seeds (different initial values for learning); bars indicate means of those 5 simulations. (*B*) Dissimilarity matrix between DSI representation vectors for 100 words in 10 semantic categories (DSI-decorr). We selected 10 words in each category. We used same dissimilarity metric with Reber et al. (32) (1 − Pearson’s correlation coefficient). (*C*) Visualization of the representational structure of DSI using MDS based on the dissimilarity matrix shown in (*B*). Each color corresponds to a semantic category.

Concept cells are considered to represent semantic relationships between concepts at the level of population activity patterns (31). Correspondingly, DSI vectors also represent the semantic structure of words because representation vectors of word embedding models have such property (36–39). We evaluated how word similarity is captured by cosine similarity of DSI vectors using the evaluation procedure in NLP (36–39). We calculated cosine similarity between representation vectors of word pairs and evaluated the rank correlation between those cosine similarities and human word similarities [WS353 dataset (70); 248/345 word pairs were used]. As a result, DSI showed high correlations that are comparable to word embedding models (Fig. 4*A*). This indicates that DSI captures the semantic structure of words at the level of population activity patterns. This property is preserved across settings (dimensionality and datasets); however, large γ degraded the correlations (*SI Appendix*, Figs. S6–S8).

This property suggests that the representational structure of DSI vectors is consistent with the experimental observation that population-level pattern similarity of concept cell activities represents semantic categories (32). We considered 10 semantic categories and chose 10 words corresponding to each category (see *SI Appendix*, *Extended Methods “Evaluation of the semantic structure of DSI vectors”* for the choice of words) and evaluated dissimilarity between DSI representation vectors of those 100 words using the same metric with the experimental work (32) (1 − Pearson’s correlation coefficient). Dissimilarity matrix in Fig. 4*B* and *SI Appendix*, Fig. S9 clearly shows that representations were similar within each semantic category. In the visualization of the representational structure by multidimensional scaling (MDS), we can also see clustering of words corresponding to 10 semantic categories (Fig. 4*C* and *SI Appendix*, Fig. S9).

## Analogical Inference by Partial Recombination of Assemblies of Conceptual Units.

**Fig. 5.**
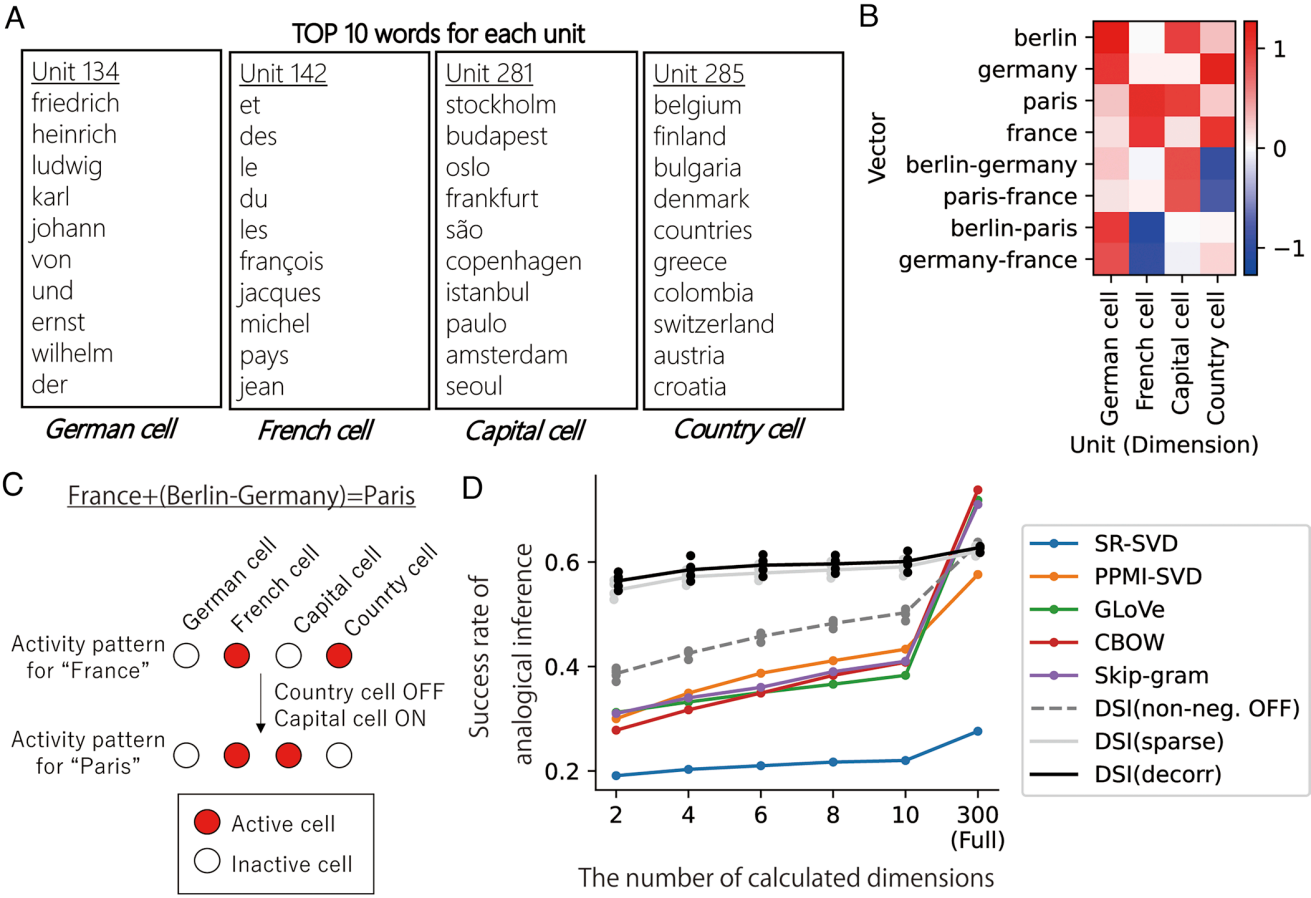
Analogical inference as a sparse recombination of conceptual units. (*A*) TOP-10 words of four representative units obtained by DSI-decorr and their interpretation. (*B*) Values of four representative units in DSI vectors for France, Germany, Paris, Berlin, and difference vectors between them. (*C*) Interpretation of analogical inference as sparse recombination of concept cells. (*D*) Success rates of analogical inference task (Mikolov’s dataset) by calculating the limited number of dimensions in word representation vectors. For DSI, dots indicate 5 trials with different random seeds (different initial values for learning); lines indicate means of those 5 simulations. Success rates of DSI-decorr were not significantly different from DSI-sparse (*P* > 0.05, 2-sample *t* test) but higher than all other methods in the condition of 2, 4, 6, 8, and 10 dimensions. (*P* < 0.01, 2-sample *t* test for DSI (non-neg. OFF) and 1-sample *t* tests for other word embedding methods). All statistical tests were two-sided *t* tests and significance thresholds were modified by Bonferroni correction. Details of statistical analyses are shown in *SI Appendix*, Table S2.

So far, we have analyzed how DSI represents spaces and semantic concepts. In this section, we discuss how DSI can support computation of semantic concepts. Specifically, we argue that DSI provides a biological interpretable mechanism for inference of words. Previous studies in NLP (36, 37) have shown that arithmetic calculation of word representation vectors enables inference of words based on analogical relationships. For example, when we consider an analogical relationship “man is to woman as king is to queen,” the calculation of word representation vectors as $\vec{x}\left(king\right)+\vec{x}\left(woman\right)-\vec{x}\left(man\right)$ gives a similar vector to $\vec{x}\left(queen\right)$. For this analogical inference task, DSI vectors show comparable performance with conventional word embedding models [evaluation by Mikolov’s dataset (36, 37); 3,157/19,544 questions were used; see *SI Appendix*, *Extended Methods “Analogical inference of words”* for details] [Fig. 5*D*, success rates at “300 (Full)”]. However, unlike the conventional word embedding, each word is represented by a combination of concept-specific units (a population activity pattern of concept cells) in DSI. In this case, we can interpret its analogical inference as a partial recombination of those assemblies to create novel word representations. Below, we give an intuitive explanation of this property of DSI.

First, we analyzed the ratio of each element to the sum of all elements in DSI vectors. We found that even the largest element accounted for 5% of the sum of all elements on average in the case of DSI-decorr (*SI Appendix*, Fig. S10). This implies that DSI vectors for words are nonsparse and distributed; thus, each word is represented by a combination of multiple conceptual units.

Next, we inspected representations of a set of words as an example: France, Paris, Germany, and Berlin. In those words, we can see two analogical relationships (country–capital and French–German relationships). We identified the most active units in DSI vectors for those words and listed the TOP-10 words for identified units. We could find four representative units that we could name as “German cell,” “French cell,” “country cell,” and “capital cell” (Fig. 5*A*). We could see that $\vec{x}\left(France\right)$ is represented by the combination of the French cell and the country cell, whereas $\vec{x}\left(Berlin\right)$ is represented by the combination of the German cell and the capital cell, and so on (Fig. 5*B*). This example gives a simple interpretation of word similarity in the DSI vector space discussed in the previous section. If words are similar, they share a large number of active conceptual units, like the country cell shared by representations of France and Germany. Thus, semantic similarity between words increases cosine similarity between word vectors.

Then, the difference vectors between words correspond to switching on and off conceptual units. For example, summing the difference vector of $\vec{x}\left(Germany\right)$ and $\vec{x}\left(Berlin\right)$ can turn on the capital cell and turn off the country cell (Fig. 5*B*). Similarly, transformation from $\vec{x}\left(France\right)$ to $\vec{x}\left(Paris\right)$ corresponds to the activation of the capital cell and deactivation of the country cell, enabling this transformation by summing the difference of $\vec{x}\left(Germany\right)$ and $\vec{x}\left(Berlin\right)$. Thus, we can biologically interpret the procedure of analogical inference as a partial recombination of an assembly of concept cells (Fig. 5*C*). If neural circuits in HPC and EC have learned transformation of $\vec{x}\left(Germany\right)$ to $\vec{x}\left(Berlin\right)$ by sparse switching of concept cells, the same operation enables $\vec{x}\left(France\right)$ to $\vec{x}\left(Paris\right)$. Here, the mathematical mechanism of the analogical inference is the same as the conventional word embedding models. However, a unique feature of our DSI representations is that those analogical relationships are factorized into separated units, making the modulation of a few units enough to perform the inference. We speculate that constraints in our dimension reduction method (especially nonnegativity) are sufficient conditions to align each semantic factor to each axis of the vector space.

Next, we check whether the property of DSI mentioned above is general across a wide repertoire of words. If each analogical relationship is actually confined to a specific dimension, switching on and off only a few dimensions is enough for the inference, and the calculation of a whole vector is unnecessary. We tested this hypothesis by an analogical inference task using Mikolov’s dataset, limiting the number of calculated dimensions. Namely, when we calculated $\vec{x}\left(France\right)+\vec{x}\left(Berlin\right)-\vec{x}\left(Germany\right)$ to obtain $\vec{x}\left(Paris\right)$, we identified a few dimensions that had maximum and minimum values in $\vec{x}\left(Berlin\right)-\vec{x}\left(Germany\right)$ (that are “country cell” and “capital cell”). We summed only those dimensions to $\vec{x}\left(France\right)$. This partial calculation of vectors did not degrade the overall task performance of DSI-decorr and DSI-sparse (even 2 out of 300 dimensions did not largely alter the performance) (Fig. 5*D*). In contrast, the performance of other word embedding models was significantly impaired (Fig. 5*D*). This result suggests that the partial recombination of concept-specific units enabled the analogical inference of various words, and this property is unique to the DSI model. Removal of the nonnegativity constraint significantly impaired the performance of DSI-decorr, which further suggests the importance of nonnegativity (Fig. 5*D*). We observed qualitatively the same results across settings (dimensionality and datasets) although the effect of nonnegativity disappeared with large γ (γ=0.99) (*SI Appendix*, Figs. S6–S8). Notably, CBOW model, which showed high conceptual specificity (Fig. 3), did not work properly in this inference task with partial calculations (Fig. 5*D*). This suggests that apparent conceptual specificity is not a sufficient condition for such calculation.

## Analogical Inference of Spatial Contexts by Modulating Nongrid Spatial Representations.

In the previous section, we discussed analogical inference of words. We found that we can apply the same computational framework to spatial representations. Furthermore, our results suggest a computational role of nongrid (heterogenous) spatial representations that have been experimentally found in EC (40, 56).

**Fig. 6.**
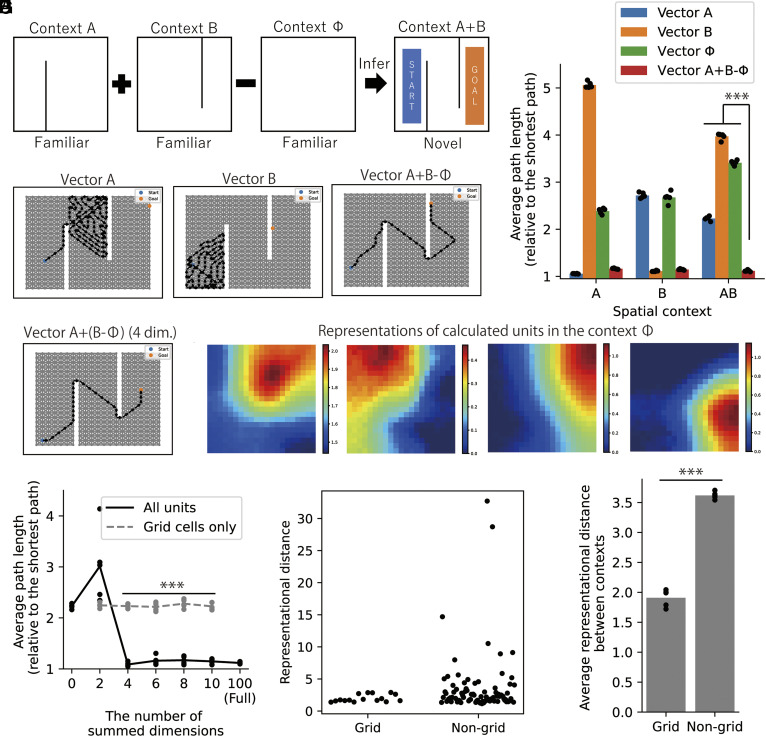
Composite spatial representations enable navigation in a novel spatial context. (*A*) We constructed representation vectors for a novel context A+B by arithmetic composition of DSI representation vectors for three familiar contexts A, B, and Φ. The start and the goal in each navigation trial were randomly positioned in the colored area. (*B*) Example spatial paths by spatial navigation using representation vectors learned in the context A (*Left*), B (*Middle*), and composite representation vectors A+B−Φ (*Right*). (*C*) Average path lengths in 1,000 trials of spatial navigation under various settings of representation vectors and contexts. Note that we normalized a path length by the shortest path length between the start and the goal in each trial. Dots indicate 5 simulations with different random seeds (different initial values for learning and simulations); bars indicate means of those 5 simulations. (*D*) An example of spatial navigation performed by representation vectors created by calculating only 4 dimensions of the vector A. (*E*) Average path lengths by the spatial navigation using the composite representation vectors in which we summed the given number of dimensions. Dots indicate 5 simulations with different random seeds (different initial values for learning and simulations); lines indicate means of those 5 simulations. 2-sample *t* tests were performed between the condition in which we calculated only grid cells and the condition in which we calculated all units. (*F*) Spatial representations of four calculated units in the context Φ in an example of (*D*). Note that all these units are nongrid representations. (*G*) Representational distances between the context B and Φ of grid-type and non-grid-type units in a simulation. Each dot indicates a unit. (*H*) Average representational distances between the context B and Φ of grid-type and non-grid-type units. Dots indicate 5 simulations with different random seeds (different initial values for learning and simulations); bars indicate means of those 5 simulations. ***P < 0.001. All statistical tests were two-sided *t* tests and significance thresholds were modified by Bonferroni correction. Details of statistical analyses are shown in *SI Appendix*, Table S3.

First, we constructed DSI representation vectors (DSI-decorr) for spatial contexts A, B, and Φ, which have different barrier layouts (Fig. 6*A* and *SI Appendix*, Fig. S11). Then, we created composite representation vectors for a novel context A+B by simply combining the spatial representation vectors for familiar contexts A, B, and Φ as “A+B−Φ” (Fig. 6*A*; see *SI Appendix*, *Extended Methods “Analogical inference of spatial contexts”* for details). We devised this computation based on the analogical inference of words: The relationship of Φ to B corresponds to the relationship of A to A+B (whether a barrier exists at a specific position or not). We tested goal-directed spatial navigation (as described in the previous section) in three spatial contexts A, B, and A+B, by using the learned representations for A, B, and Φ, and those generated for A+B. Naturally, the representation vectors for A and B gave the best performance in contexts A and B, respectively (Fig. 6 *B* and *C*). Remarkably, the composite representation vector “A+B−Φ” achieved the best performance in the context A+B (Fig. 6 *B* and *C*). This result corresponds to the vector computation for word inference, suggesting that the computational framework for semantic concepts is also valid for inferring the spatial contexts. We confirmed that this inference also worked in another spatial setting (*SI Appendix*, Fig. S12). However, the inference did not improve spatial navigation when two barriers were spatially connected in the context A+B (*SI Appendix*, Fig. S13). We speculate that this simple computational scheme works only if the interaction between barriers is small. Therefore, we conducted further analyses below in the settings used in Fig. 6 and *SI Appendix*, Fig. S12.

We hypothesized that the relationship between spatial contexts is confined to the small number of units (dimensions) as in word representations. If this is the case, representational modulations of only a few units are enough for the inference. We identified a few units that had the largest representational differences (see *SI Appendix*, *Extended Methods “Analogical inference of spatial contexts”* for the definition of representational distance) between the spatial contexts B and Φ, and calculated only those units to compose representation vectors for the context A+B. We found that minimally 4 units were enough to obtain the asymptotic performance (Fig. 6 *D* and *E*). We inspected the spatial representations of these units and found that all of them were nongrid representations in the context Φ (no barriers) (Fig. 6*F*). Consistently, we found large representational distances selectively in non-grid-type units (Fig. 6*G*), and the average representational distance of non-gird-type units is larger than that of grid-type units (Fig. 6*H* and *SI Appendix*, Fig. S12). Strikingly, when we restricted calculation of the vectors to grid-type units (excluding non-grid-type units), the obtained composite representation vectors did not improve spatial navigation in the context A+B (Fig. 6*E*). We obtained qualitatively same results in the other spatial setting (*SI Appendix*, Fig. S12). These results suggest that information of spatial contexts (barrier settings in this case) is confined to the limited number of units that have nongrid spatial representations, and the modulation of nongrid representations is sufficient for inferring novel spatial contexts. We could obtain qualitatively same results when the dimensionality of representation vectors was changed from D = 100 to D = 50 or D = 150 (*SI Appendix*, Fig. S14).

These results imply that the modulation of nongrid spatial representations is more informative for coding differences between spatial contexts than that of grid representations. This is consistent with the previous experimental finding that nongrid spatial representations are remapped more significantly than grid cells across different spatial contexts (40). Our model provides a theoretical interpretation for this experimental observation, linking the computation of spatial contexts to that of semantic concepts.

## Discussion

In this paper, we proposed a theoretically interpretable and biologically plausible neural representation model for spatial navigation and semantic concepts. Our model is mathematically related to reinforcement learning and word embedding, thus representations support spatial navigation and NLP. We demonstrated that our DSI model forms spatially local or hexagonal grid representations for the 2-D space and concept-specific representations for the linguistic inputs, which can be regarded as neural representations in HPC and EC. Finally, the same computational framework based on partial representational modulation enables the inference of words and spatial contexts, which can be biologically interpreted as the function of concept cells and nongrid cells. These results suggest that we can extend the spatial representation model of HPC and EC to learn and compute semantic concepts, which apparently seems a different computational domain from spatial navigation, in an intuitive and biologically plausible manner.

### Theory of Grid and Nongrid Representations in EC.

Our model produces grid-like representations in 2-D space, which supports path integration and spatial navigation. Previous studies have revealed that nonnegative and orthogonal constraints are important to obtain realistic grid-like representations (41, 42, 60). Furthermore, recurrent neural networks form grid-like representations through learning path integration, and those representations support efficient spatial navigation (42–46). It has also been shown that a unified model for spatial and nonspatial cognition generates grid representations (52, 53, 55). Although our model was built on the basis of those previous works, previous models have not been applied to learning of semantic concepts or other complex conceptual spaces in real-world data. Furthermore, our results revealed that the computational framework for semantic concepts (analogical inference) can be applied to spatial representations, and nongrid spatial representations were important for the inference. Our model extended the range of applicability of biologically plausible spatial representation models to semantic concepts and suggests shared computational mechanism between those two computational domains.

Furthermore, our model suggests the importance of nongrid spatial representations for inference (or switching) of spatial contexts. In EC, there are many neurons that exhibit nongrid spatial representations, and they show large remapping across contexts (40, 56). Previous models have shown that nongrid (heterogenous) spatial representations in EC support precise spatial encoding (42) and reward-based modulation of path integration (46). Our results give alternative explanation of such remapping: Such remapping can support inference of navigation strategy across spatial contexts. However, it is still to be investigated whether this computational framework extends to general contextual changes such as wall colors, odors, and reward settings (40, 46, 56). We speculate that this is possible if we can appropriately construct disentangled representations for general sensory and task variables (61, 71, 72).

### Implications for Conceptual Representations in the Brain.

Models of concept cells have mostly assumed sparsity and clustering to create conceptual specificity (47, 48, 52, 53, 58). In contrast, our results suggest that nonnegativity is essential to create functionally useful concept-specific representations. Nonnegativity is also crucial to generate biologically plausible grid representations (41, 42, 60), thus our model suggests a common constraint across spatial and conceptual representations in HPC and EC. A recent theoretical finding suggests that nonnegativity and energy minimization enable disentanglement of visual, spatial, and task variables from mixed inputs (61) (also see *SI Appendix*, *Extended Discussion “Relationships with disentangled visual representation learning”*). Our results suggest that such strategy also works in the case of semantic concepts, and it enables a simple framework for the inference in the brain. Although it has already been known that nonnegativity is useful for representation learning (41, 42, 58, 60, 61, 73), we propose that nonnegativity also contributes to the formation of concept cells from complex sensory inputs like word sequences, and the underlying mechanism is shared with spatial representation learning in the same brain regions. It is also notable that our model predicts population-level properties of concept cells in addition to representations in the unit level (*SI Appendix*, *Extended Discussion “Prediction for concept cells in the population level”*).

In contrast to nonnegativity, switching decorrelation and sparsity (corresponding to representations for EC and HPC, respectively) did not make qualitative differences in functionality of conceptual representations in our evaluations. Consistently with our results, similar concept cells exist in both EC and HPC in the human brain (31, 32). However, further studies may reveal functional differences between those constraints (*SI Appendix*, *Extended Discussion “Possible functional difference between EC and HPC”*). We further discussed roles of nonnegativity and decorrelation in our model in *SI Appendix*, *Extended Discussion “Roles of constraints in DSI.”*

### Relationships with Hippocampal Memory Function.

Further studies should clarify whether DSI can explain a wider range of hippocampal memory functions. Free recall of given words is often used to evaluate memory functions in psychology (74, 75), and the underlying process has been modeled by Hopfield-type attractor neural network models (76–78). In these models, memory recall is interpreted as a transition across neural activity patterns embedded as attractors, and transition probabilities between these attractors are positively correlated with pattern similarity. Because DSI representations of two words become similar when the words share a semantic similarity, combining DSI with attractor network models likely enables us to investigate the relationship between semantic structures and the memory recall process. For example, such a model may explain the creation of false memory which is semantically similar to actually memorized items (74). Furthermore, the combination of recently learned associations in the hippocampus enables inference of novel relationships (79). An online extension of DSI may enable such inference based on one-shot memories.

Recently, memory formation and consolidation in the corticohippocampal system were modeled as generative models, specifically variational autoencoder (80, 81). One of these models enabled an arithmetic computation of visual semantic concepts like our model (81). We may reconcile DSI with those generative models by extending immediate inference to future prediction. We may use such models to elucidate how predictive hippocampal representations are useful for forming semantic representations in high-order sensory cortical areas, and how hippocampal memory is employed for decision making.

### Biological Interpretations of the DSI Model.

In this paper, we derived our model from theoretical perspectives. Here, we discuss biological interpretations and implementations of the model. First, in our model, the performance strongly depends on the setting of the parameter γ, which corresponds to the temporal discount rate for successor representation and determines the timescale of prediction (*SI Appendix*, Figs. S2 and S6–S8). Therefore, the biological plausibility of the setting of γ is essential to ground our model to the actual brain. We quantitatively estimate that the setting of γ for DSI is biologically possible under the assumption that SR-like neural representations are created by behavioral time scale synaptic plasticity (82) and a gradient of representational timescales (83) exists in the hippocampus (*SI Appendix*, *Extended Discussion “Biological plausibility of the timescale of prediction in the model”*). Second, how biological neural networks can learn DSI is unclear. We speculate that we can build a hippocampal neural network model for DSI by extending the model for word embedding (*SI Appendix*, *Extended Discussion “Possible biological implementations of DSI”*). Third, although we interpreted our model as a model of HPC and EC, we can also interpret the units in our model as “semantic features” (attributes) in a broad sense (49–51, 84–86) and the model may be related to other brain regions such as anterior temporal lobe (ATL) (49, 85) and prefrontal cortex (PFC) (8–10, 87, 88) (*SI Appendix*, *Extended Discussion “Alternative biological interpretations of DSI”*). In addition, we note that the role of HPC and EC in spatial computations is still debatable and needs further validation (*SI Appendix*, *Extended Discussion “The role of HPC and EC in spatial tasks”*). These possibilities of biological interpretation and implementation should be investigated in future studies.

### Neural Mechanism for Natural Language Processing in the Brain.

Our model relates NLP, especially word embedding to conceptual representations in HPC and EC. Concept cells respond to words (both auditory inputs and texts) (31, 33), and the relationship between HPC and language processing has been experimentally found (89, 90). Regarding word embedding, a previous study showed that hippocampal theta oscillation codes semantic distances between words measured in word2vec (skip-gram) subspace (91). These experimental results suggest the possible relationship between HPC and semantic processing for language. However, the contributions of HPC and EC to language processing are still controversial and require further validation. It is also possible that HPC and EC are important only in the initial stage of learning novel semantic information considering the memory consolidation hypothesis (92). In this case, HPC and EC may not be necessary for using familiarized languages.

Recent studies have shown that representations in transformer-based models (93) such as GPT (94) achieve remarkable performance in linear fitting to neural recording during language processing (95, 96). Furthermore, it was recently shown that transformer-based models generate grid-like representations when applied to spatial learning (54). Similarly to our model, this finding implies the relationship between spatial and linguistic processing in the brain although concept-specific representations have not been found in transformer-based models. A major difference between our DSI model and transformer-based models is that DSI representations are basically fixed (static embedding) whereas transformer-based models flexibly create context-dependent representations (dynamic embedding). Computation of concepts obviously depends on the context; thus activities of concept cells are dependent on contexts such as memory contents and task demands (33). Therefore, our DSI model should be extended to process context dependence, hopefully by combination with other models for learning context-dependent latent cognitive states (55, 97, 98).

Among transformer-based models, predictive (asymmetric) models (GPT) show better fits to the neural responses to linguistic inputs than bidirectional (symmetric) models (such as BERT) (95, 96). This suggests that future prediction drives the emergence of semantic representations in the brain, which is consistent with the claim of our model. We speculate that asymmetric predictive representations are useful for value evaluation for decision making, and also for generating forward sequences observed in the hippocampus which in turn help path planning in spatial navigation (99) and generating word/event sequences like GPT.

## Materials and Methods

We provide a summary of our methods, with further details described in *SI Appendix*, *Extended Methods*. We performed dimension reduction for DSI-decorr by the gradient descent of an objective function that consists of square errors between $PSI\left(s,s^{\prime }\right)$ and $\vec{x}\left(s\right)\cdot \vec{w}\left(s^{\prime }\right)$, squared correlations between pairs of dimensions of $\vec{x}\left(s\right)$, and L2 norms of $\vec{x}\left(s\right)$ and $\vec{w}\left(s\right)$. For DSI-sparse, we removed the decorrelation term and changed L2 norms to L1 norms. To quantify the conceptual specificity of each unit, we obtained WordNet-based semantic similarity of TOP-10 words and 1,000 randomly sampled word pairs (a null distribution). The unit was classified as a concept-specific unit if the mean similarity of TOP-10 words exceeded 95 percentile of the null distribution, and the conceptual specificity was calculated as $\frac{s_{\mathrm{unit}}}{s_{\mathrm{null}}}-1$ where $s_{\mathrm{unit}}$ is the mean similarity of TOP-10 words and $s_{\mathrm{null}}$ is the mean of the null distribution. In the experiment of analogical inference of spatial contexts, we constructed representation vectors for contexts A, B, Ф through direct experiences, then we created new vectors as $\vec{x}\left(s_{i}^{A+B}\right)=\vec{x}\left(s_{i}^{A}\right)+\vec{x}\left(s_{i}^{B}\right)-\vec{x}\left(s_{i}^{\Phi }\right)$, where $s_{i}^{A},\;s_{i}^{B},\;s_{i}^{\Phi }$ and $s_{i}^{A+B}$ are states in contexts A, B, Ф, A+B, and i is a positional index which indicates a same position in all contexts. The representational distance of the unit k [k-th dimension of vectors $x_{k}\left(s\right)$] was defined as $\sum_{i}{\left(x_{k}\left(s_{i}^{B}\right)-x_{k}\left(s_{i}^{\Phi }\right)\right)}^{2}$.

## Supplementary Material

Appendix 01 (PDF)

## Data Availability

Python codes for simulations and analyses are available at https://github.com/TatsuyaHaga/DSI_codes. We also used external codes at https://github.com/stanfordnlp/GLoVe (GLoVe) and https://github.com/attardi/wikiextractor (wikiextrator). Text data were taken from https://dumps.wikimedia.org/enwiki/latest/ (version 22-May-2020). We also used WikiText-103 dataset (69). We share the preprocessed datasets we used at https://zenodo.org/records/11651117 under CC-BY-SA license. WS353 dataset is available at http://alfonseca.org/eng/research/wordsim353.html. Mikolov’s dataset is available at https://aclweb.org/aclwiki/Google_analogy_test_set_(State_of_the_art).

## References

[1] T. Hafting, M. Fyhn, S. Molden, M. B. Moser, E. I. Moser, Microstructure of a spatial map in the entorhinal cortex. Nature **436**, 801–806 (2005). 15965463 10.1038/nature03721

[2] C. F. Doeller, C. Barry, N. Burgess, Evidence for grid cells in a human memory network. Nature **463**, 657–661 (2010). 20090680 10.1038/nature08704 PMC3173857

[3] J. Jacobs , Direct recordings of grid-like neuronal activity in human spatial navigation. Nat. Neurosci. **16**, 1188–1190 (2013). 23912946 10.1038/nn.3466 PMC3767317

[4] J. O’Keefe, J. Dostrovsky, The hippocampus as a spatial map. Preliminary evidence from unit activity in the freely-moving rat. Brain Res. **34**, 171–175 (1971). 5124915 10.1016/0006-8993(71)90358-1

[5] D. Bush, C. Barry, D. Manson, N. Burgess, Using grid cells for navigation. Neuron **87**, 507–520 (2015). 26247860 10.1016/j.neuron.2015.07.006 PMC4534384

[6] B. L. McNaughton, F. P. Battaglia, O. Jensen, E. I. Moser, M. B. Moser, Path integration and the neural basis of the “cognitive map”. Nat. Rev. Neurosci. **7**, 663–678 (2006). 16858394 10.1038/nrn1932

[7] Y. Burak, I. R. Fiete, Accurate path integration in continuous attractor network models of grid cells. PLoS Comput. Biol. **5**, e1000291 (2009). 19229307 10.1371/journal.pcbi.1000291 PMC2632741

[8] A. O. Constantinescu, J. X. O’reilly, T. E. J. Behrens, Organizing conceptual knowledge in humans with a gridlike code. Science **352**, 1464–1468 (2016). 27313047 10.1126/science.aaf0941 PMC5248972

[9] X. Bao , Grid-like neural representations support olfactory navigation of a two-dimensional odor space. Neuron **102**, 1066–1075.e5 (2019). 31023509 10.1016/j.neuron.2019.03.034 PMC7497729

[10] S. A. Park, D. S. Miller, E. D. Boorman, Inferences on a multidimensional social hierarchy use a grid-like code. Nat. Neurosci. **24**, 1292–1301 (2021). 34465915 10.1038/s41593-021-00916-3 PMC8759596

[11] S. Viganò, M. Piazza, Distance and direction codes underlie navigation of a novel semantic space in the human brain. J. Neurosci. **40**, 2727–2736 (2020). 32060171 10.1523/JNEUROSCI.1849-19.2020 PMC7096136

[12] S. Viganò, V. Rubino, A. Di Soccio, M. Buiatti, M. Piazza, Grid-like and distance codes for representing word meaning in the human brain. Neuroimage **232**, 117876 (2021). 33636346 10.1016/j.neuroimage.2021.117876

[13] S. Theves, G. Fernandez, C. F. Doeller, The hippocampus encodes distances in multidimensional feature space. Curr. Biol. **29**, 1226–1231.e3 (2019). 30905602 10.1016/j.cub.2019.02.035

[14] D. Aronov, R. Nevers, D. W. Tank, Mapping of a non-spatial dimension by the hippocampal-entorhinal circuit. Nature **543**, 719–722 (2017). 28358077 10.1038/nature21692 PMC5492514

[15] E. B. Knudsen, J. D. Wallis, Hippocampal neurons construct a map of an abstract value space. Cell **184**, 4640–4650.e10 (2021). 34348112 10.1016/j.cell.2021.07.010 PMC8459666

[16] W. Schultz, P. Dayan, P. R. Montague, A neural substrate of prediction and reward. Science **275**, 1593–1599 (1997). 9054347 10.1126/science.275.5306.1593

[17] M. M. Botvinick, Hierarchical reinforcement learning and decision making. Curr. Opin. Neurobiol. **22**, 956–962 (2012). 22695048 10.1016/j.conb.2012.05.008

[18] M. M. Botvinick, Y. Niv, A. C. Barto, Hierarchically organized behavior and its neural foundations: A reinforcement learning perspective. Cognition **113**, 262–280 (2009). 18926527 10.1016/j.cognition.2008.08.011 PMC2783353

[19] H. Makino, Arithmetic value representation for hierarchical behavior composition. Nat. Neurosci. **26**, 140–149 (2023). 36550292 10.1038/s41593-022-01211-5 PMC9829535

[20] L. Cross, J. Cockburn, Y. Yue, J. P. O’Doherty, Using deep reinforcement learning to reveal how the brain encodes abstract state-space representations in high-dimensional environments. Neuron **109**, 724–738.e7 (2021). 33326755 10.1016/j.neuron.2020.11.021 PMC7897245

[21] M. G. Mattar, N. D. Daw, Prioritized memory access explains planning and hippocampal replay. Nat. Neurosci. **21**, 1609–1617 (2018). 30349103 10.1038/s41593-018-0232-z PMC6203620

[22] P. Dayan, Improving generalization for temporal difference learning: The successor representation. Neural Comput. **5**, 613–624 (1993).

[23] E. M. Russek, I. Momennejad, M. M. Botvinick, S. J. Gershman, N. D. Daw, Predictive representations can link model-based reinforcement learning to model-free mechanisms. PLoS Comput. Biol. **13**, e1005768 (2017). 28945743 10.1371/journal.pcbi.1005768 PMC5628940

[24] K. L. Stachenfeld, M. M. Botvinick, S. J. Gershman, The hippocampus as a predictive map. Nat. Neurosci. **20**, 1643–1653 (2017). 28967910 10.1038/nn.4650

[25] I. Momennejad, Learning structures: Predictive representations, replay, and generalization. Curr. Opin. Behav. Sci. **32**, 155–166 (2020). 35419465 10.1016/j.cobeha.2020.02.017 PMC9004662

[26] I. Momennejad , The successor representation in human reinforcement learning. Nat. Hum. Behav. **1**, 680–692 (2017). 31024137 10.1038/s41562-017-0180-8 PMC6941356

[27] M. M. Garvert, R. J. Dolan, T. E. Behrens, A map of abstract relational knowledge in the human hippocampal–entorhinal cortex. Elife **6**, e17086 (2017). 28448253 10.7554/eLife.17086 PMC5407855

[28] E. Todorov, “Linearly-solvable Markov decision problems” in Advances in Neural Information Processing Systems 19, B. Schölkopf, J. Platt, T. Hoffman, Eds. (MIT Press, Cambridge, MA, 2006), pp. 1369–1376.

[29] E. Todorov, Efficient computation of optimal actions. Proc. Natl. Acad. Sci. U.S.A. **106**, 11478–11483 (2009). 19574462 10.1073/pnas.0710743106 PMC2705278

[30] P. Piray, N. D. Daw, Linear reinforcement learning in planning, grid fields, and cognitive control. Nat. Commun. **12**, 4942 (2021). 34400622 10.1038/s41467-021-25123-3 PMC8368103

[31] R. Q. Quiroga, Concept cells: The building blocks of declarative memory functions. Nat. Rev. Neurosci. **13**, 587–597 (2012). 22760181 10.1038/nrn3251

[32] T. P. Reber , Representation of abstract semantic knowledge in populations of human single neurons in the medial temporal lobe. PLoS Biol. **17**, e3000290 (2019). 31158216 10.1371/journal.pbio.3000290 PMC6564037

[33] M. Bausch , Concept neurons in the human medial temporal lobe flexibly represent abstract relations between concepts. Nat. Commun. **12**, 6164 (2021). 34697305 10.1038/s41467-021-26327-3 PMC8545952

[34] G. Kreiman, C. Koch, I. Fried, Imagery neurons in the human brain. Nature **408**, 357–361 (2000). 11099042 10.1038/35042575

[35] H. Gelbard-Sagiv, R. Mukamel, M. Harel, R. Malach, I. Fried, Internally generated reactivation of single neurons in human hippocampus during free recall. Science **322**, 96–101 (2008). 18772395 10.1126/science.1164685 PMC2650423

[36] T. Mikolov, K. Chen, G. Corrado, J. Dean, “Distributed representations of words and phrases and their compositionality” in Advances in Neural Information Processing Systems 26, C. J. Burges, L. Bottou, M. Welling, Z. Ghahramani, K. Q. Weinberger, Eds. (Curran Associates, Red Hook, NY, 2013).

[37] T. Mikolov, K. Chen, G. Corrado, J. Dean, Efficient estimation of word representations in vector space. arXiv [Preprint] (2013). 10.48550/arXiv.1301.3781 (Accessed 31 August 2021).

[38] J. Pennington, R. Socher, C. Manning, “Glove: Global vectors for word representation” in Proceedings of the 2014 Conference on Empirical Methods in Natural Language Processing (EMNLP), A. Moschitti, B. Pang, W. Daelemans, Eds. (Association for Computational Linguistics, 2014), pp. 1532–1543.

[39] O. Levy, Y. Goldberg, “Neural word embedding as implicit matrix factorization” in Advances in Neural Information Processing Systems 7, Z. Ghahramani, M. Welling, C. Cortes, N. Lawrence, K. Q. Weinberger, Eds. (Curran Associates, Inc., Red Hook, NY, 2005), pp. 2177–2185.

[40] G. W. Diehl, O. J. Hon, S. Leutgeb, J. K. Leutgeb, Grid and nongrid cells in medial entorhinal cortex represent spatial location and environmental features with complementary coding schemes. Neuron **94**, 83–92.e6 (2017). 28343867 10.1016/j.neuron.2017.03.004 PMC5444540

[41] Y. Dordek, D. Soudry, R. Meir, D. Derdikman, Extracting grid cell characteristics from place cell inputs using non-negative principal component analysis. Elife **5**, e10094 (2016), 10.7554/eLife.10094.001. 26952211 PMC4841785

[42] B. Sorscher, G. C. Mel, S. A. Ocko, L. M. Giocomo, S. Ganguli, A unified theory for the computational and mechanistic origins of grid cells. Neuron **111**, 121–137.e13 (2023). 36306779 10.1016/j.neuron.2022.10.003

[43] A. Banino , Vector-based navigation using grid-like representations in artificial agents. Nature **557**, 429–433 (2018).29743670 10.1038/s41586-018-0102-6

[44] C. J. Cueva, X.-X. Wei, “Emergence of grid-like representations by training recurrent neural networks to perform spatial localization” in International Conference on Learning Representations (ICLR, 2018).

[45] R. Gao, J. Xie, S.-C. Zhu, Y. N. Wu, “Learning grid cells as vector representation of self-position coupled with matrix representation of self-motion” in International Conference on Learning Representations (ICLR, 2019).

[46] A. Nayebi , “Explaining heterogeneity in medial entorhinal cortex with task-driven neural networks” in Advances in Neural Information Processing Systems, M. Ranzato, A. Beygelzimer, Y. Dauphin, P. S. Liang, J. Wortman Vaughan, Eds. (Curran Associates, Red Hook, NY, 2005), pp. 12167–12179.

[47] S. Waydo, C. Koch, Unsupervised learning of individuals and categories from images. Neural Comput. **20**, 1165–1178 (2008). 18194101 10.1162/neco.2007.03-07-493

[48] C. Calvo Tapia, I. Tyukin, V. A. Makarov, Universal principles justify the existence of concept cells. Sci. Rep. **10**, 7889 (2020). 32398873 10.1038/s41598-020-64466-7 PMC7217959

[49] M. A. L. Ralph, E. Jefferies, K. Patterson, T. T. Rogers, The neural and computational bases of semantic cognition. Nat. Rev. Neurosci. **18**, 42–55 (2017). 27881854 10.1038/nrn.2016.150

[50] T. T. Rogers, J. L. McClelland, Précis of semantic cognition: A parallel distributed processing approach. Behav. Brain Sci. **31**, 689–714 (2008).

[51] A. M. Saxe, J. L. McClelland, S. Ganguli, A mathematical theory of semantic development in deep neural networks. Proc. Natl. Acad. Sci. U.S.A. **166**, 11537–11546 (2019). 10.1073/pnas.1820226116 PMC6561300 31101713

[52] R. M. Mok, B. C. Love, A multilevel account of hippocampal function in spatial and concept learning: Bridging models of behavior and neural assemblies. Sci. Adv. **9**, eade6903 (2023). 37478189 10.1126/sciadv.ade6903 PMC10361583

[53] R. M. Mok, B. C. Love, A non-spatial account of place and grid cells based on clustering models of concept learning. Nat. Commun. **10**, 5685 (2019). 31831749 10.1038/s41467-019-13760-8 PMC6908717

[54] J. C. R. Whittington, J. Warren, T. E. J. Behrens, “Relating transformers to models and neural representations of the hippocampal formation” in International Conference on Learning Representations (ICLR, 2022).

[55] J. C. R. Whittington , The Tolman-Eichenbaum machine: Unifying space and relational memory through generalization in the hippocampal formation. Cell **183**, 1249–1263.e23 (2020). 33181068 10.1016/j.cell.2020.10.024 PMC7707106

[56] X. Huang , Distinct spatial maps and multiple object codes in the lateral entorhinal cortex. Neuron **111**, 3068–3083.e7 (2023). 37478849 10.1016/j.neuron.2023.06.020

[57] Z. S. Harris, Distributional structure. WORD **10**, 146–162 (1954).

[58] B. Murphy, P. P. Talukdar, T. Mitchell, “Learning effective and interpretable semantic models using non-negative sparse embedding” in International Conference on Computational Linguistics (Association for Computational Linguistics, 2012), pp. 1933–1950.

[59] D. D. Lee, H. S. Seung, Learning the parts of objects by non-negative matrix factorization. Nature **401**, 788–791 (1999). 10548103 10.1038/44565

[60] B. Sorscher, G. C. Mel, S. Ganguli, S. A. Ocko, “A unified theory for the origin of grid cells through the lens of pattern formation” in Advances in Neural Information Processing Systems, H. Wallach , Eds. (Curran Asssociates, Red Hook, NY, 2019), pp. 10003–10013.

[61] J. C. R. Whittington, W. Dorrell, S. Ganguli, S. Timothy, E. J. Behrens, “Disentanglement with biological constraints: A theory of functional cell types” in The Eleventh International Conference on Learning Representations (ICLR, 2022).

[62] F. Sargolini , Conjunctive representation of position, direction, and velocity in entorhinal cortex. Science **312**, 758–762 (2006). 16675704 10.1126/science.1125572

[63] R. F. Langston , Development of the spatial representation system in the rat. Science **328**, 1576–1580 (2010). 20558721 10.1126/science.1188210

[64] C. Barry, N. Burgess, To be a Grid Cell: Shuffling procedures for determining “Gridness”. bioRxiv [Preprint] (2017). 10.1101/230250 (Accessed 26 December 2023).

[65] J. Oh, X. Guo, H. Lee, R. Lewis, S. Singh, “Action-conditional video prediction using deep networks in Atari games” in Advances in Neural Information Processing Systems, C. Cortes, N. Lawrence, D. Lee, M. Sugiyama, R. Garnett, Eds. (Curran Associates, Red Hook, NY, 2015), pp. 2863–2871.

[66] Princeton University, About Wordnet (2010). https://wordnet.princeton.edu/.

[67] J. Devlin, M.-W. Chang, K. Lee, K. Toutanova, “BERT: Pre-training of deep bidirectional transformers for language understanding” in Proceedings of the 2019 Conference of the North American Chapter of the Association for Computational Linguistics: Human Language Technologies, Volume 1 (Long and Short Papers), J. Burstein, C. Doran, T. Solorio, Eds. (Association for Computational Linguistics, 2019), pp. 4171–4186.

[68] T. Wolf , “Transformers: State-of-the-art natural language processing” in Proceedings of the 2020 Conference on Empirical Methods in Natural Language Processing: System Demonstrations (2020), pp. 38–45.

[69] S. Merity, C. Xiong, J. Bradbury, R. Socher, “Pointer sentinel mixture models” in International Conference on Learning Representations (ICLR, 2017).

[70] E. Agirre , “A study on similarity and relatedness using distributional and WordNet-based approaches” in Proceedings of Human Language Technologies: The 2009 Annual Conference of the North American Chapter of the Association for Computational Linguistics, M. Ostendorf, M. Collins, S. Narayanan, D. W. Oard, L. Vanderwende, Eds. (Association for Computational Linguistics, 2009), pp. 19–27.

[71] I. Higgins , “beta-VAE: Learning basic visual concepts with a constrained variational framework” in International Conference on Learning Representations (ICLR, 2017).

[72] I. Higgins , Unsupervised deep learning identifies semantic disentanglement in single inferotemporal face patch neurons. Nat. Commun. **12**, 6456 (2021). 34753913 10.1038/s41467-021-26751-5 PMC8578601

[73] E. Oja, M. Plumbley, Blind separation of positive sources by globally convergent gradient search. Neural Comput. **16**, 1811–1825 (2004). 15265323 10.1162/0899766041336413

[74] H. L. Roediger, K. B. Mcdermott, Creating false memories: Remembering words not presented in lists. J. Exp. Psychol.: Learn. Mem. Cogn. **21**, 803–814 (1995).

[75] L. Standing, Learning 10000 pictures. Q. J. Exp. Psychol. **25**, 207–222 (1973). 4515818 10.1080/14640747308400340

[76] S. Recanatesi, M. Katkov, S. Romani, M. Tsodyks, Neural network model of memory retrieval. Front. Comput. Neurosci. **9**, 149 (2015). 26732491 10.3389/fncom.2015.00149 PMC4681782

[77] S. Romani, I. Pinkoviezky, A. Rubin, M. Tsodyks, Scaling laws of associative memory retrieval. Neural Comput. **25**, 2523–2544 (2013). 23777521 10.1162/NECO_a_00499

[78] C. Gastaldi, T. Schwalger, E. de Falco, R. Q. Quiroga, W. Gerstner, When shared concept cells support associations: Theory of overlapping memory engrams. PLoS Comput. Biol. **17**, e1009691 (2021). 34968383 10.1371/journal.pcbi.1009691 PMC8754331

[79] D. Shohamy, A. D. Wagner, Integrating memories in the human brain: Hippocampal-midbrain encoding of overlapping events. Neuron **60**, 378–389 (2008). 18957228 10.1016/j.neuron.2008.09.023 PMC2628634

[80] Z. Fayyaz , A model of semantic completion in generative episodic memory. Neural Comput. **34**, 1841–1870 (2022). 35896150 10.1162/neco_a_01520

[81] E. Spens, N. Burgess, A generative model of memory construction and consolidation. Nat. Hum. Behav. **8**, 526–543 (2024). 38242925 10.1038/s41562-023-01799-z PMC10963272

[82] K. C. Bittner, A. D. Milstein, C. Grienberger, S. Romani, J. C. Magee, Behavioral time scale synaptic plasticity underlies CA1 place fields. Science **357**, 1033–1036 (2017). 28883072 10.1126/science.aan3846 PMC7289271

[83] B. A. Strange, M. P. Witter, E. S. Lein, E. I. Moser, Functional organization of the hippocampal longitudinal axis. Nat. Rev. Neurosci. **15**, 655–669 (2014). 25234264 10.1038/nrn3785

[84] T. M. Mitchell , Predicting human brain activity associated with the meanings of nouns. Science **320**, 1191–1195 (2008). 18511683 10.1126/science.1152876

[85] K. Patterson, P. J. Nestor, T. T. Rogers, Where do you know what you know? The representation of semantic knowledge in the human brain. Nat. Rev. Neurosci. **8**, 976–987 (2007). 18026167 10.1038/nrn2277

[86] S. Nishida, S. Nishimoto, Decoding naturalistic experiences from human brain activity via distributed representations of words. Neuroimage **180**, 232–242 (2018). 28801255 10.1016/j.neuroimage.2017.08.017

[87] E. Z. Patai, H. J. Spiers, The versatile wayfinder: Prefrontal contributions to spatial navigation. Trends Cogn. Sci. **25**, 520–533 (2021). 33752958 10.1016/j.tics.2021.02.010

[88] S. A. Bunge, C. Wendelken, D. Badre, A. D. Wagner, Analogical reasoning and prefrontal cortex: Evidence for separable retrieval and integration mechanisms. Cereb. Cortex **15**, 239–249 (2005). 15238433 10.1093/cercor/bhh126

[89] M. C. Duff, S. Brown-Schmidt, The hippocampus and the flexible use and processing of language. Front. Hum. Neurosci. **6**, 69 (2012), 10.3389/fnhum.2012.00069. 22493573 PMC3319917

[90] V. Piai , Direct brain recordings reveal hippocampal rhythm underpinnings of language processing. Proc. Natl. Acad. Sci. U.S.A. **113**, 11366–11371 (2016). 27647880 10.1073/pnas.1603312113 PMC5056038

[91] E. A. Solomon, B. C. Lega, M. R. Sperling, M. J. Kahana, Hippocampal theta codes for distances in semantic and temporal spaces. Proc. Natl. Acad. Sci. U.S.A. **116**, 24343–24352 (2019). 31723043 10.1073/pnas.1906729116 PMC6883851

[92] P. W. Frankland, B. Bontempi, The organization of recent and remote memories. Nat. Rev. Neurosci. **6**, 119–130 (2005). 15685217 10.1038/nrn1607

[93] A. Vaswani , “Attention is all you need” in Advances in Neural Information Processing Systems, I. Guyon , Eds. (Curran Associates, Red Hook, NY, 2017), pp. 5998–6008.

[94] T. B. Brown , “Language models are few-shot learners” in Advances in Neural Information Processing Systems 33, H. Larochelle, M. Ranzato, R. Hadsell, M. F. Balcan, H. Lin , Eds. (Curran Associates, Red Hook, NY, 2020), pp. 1877–1901.

[95] A. Goldstein , Shared computational principles for language processing in humans and deep language models. Nat. Neurosci. **25**, 369–380 (2022). 35260860 10.1038/s41593-022-01026-4 PMC8904253

[96] M. Schrimpf , The neural architecture of language: Integrative modeling converges on predictive processing. Proc. Natl. Acad. Sci. U.S.A. **118**, e2105646118 (2021). 34737231 10.1073/pnas.2105646118 PMC8694052

[97] B. Uria , A model of egocentric to allocentric understanding in mammalian brains. bioRxiv [Preprint] (2020). 10.1101/2020.11.11.378141 (Accessed 16 September 2022).

[98] D. George , Clone-structured graph representations enable flexible learning and vicarious evaluation of cognitive maps. Nat. Commun. **12**, 2392 (2021). 33888694 10.1038/s41467-021-22559-5 PMC8062558

[99] B. E. Pfeiffer, D. J. Foster, Hippocampal place-cell sequences depict future paths to remembered goals. Nature **497**, 74–79 (2013). 23594744 10.1038/nature12112 PMC3990408

