# Supplementary material for: A unified neural representation model for spatial and conceptual computations
Source: Proc Natl Acad Sci U S A. 2025 Mar 10;122(11):e2413449122. doi: 10.1073/pnas.2413449122 (PMC11929392; doi:10.1073/pnas.2413449122)
Supplement: Supplementary file 1 — Appendix 01 (PDF) [file pnas.2413449122.sapp.pdf]

PNAS

Supporting Information for

A unified neural representation model for spatial and semantic computations

Tatsuya Haga, Yohei Oseki, and Tomoki Fukai

Tatsuya Haga

Email: tatsuyahaga@nict.go.jp

This PDF file includes:

Supporting text (extended methods and discussion)

Figures S1 to S15

Tables S1 to S3

SI References

Extended Methods

Mathematical relationship of SI and linear reinforcement learning. In this section, we show that SI corresponds to a value function of linear reinforcement learning (1–3) in the setting of spatial navigation.

In linear reinforcement learning, an agent aims to maximize "gain" instead of reward. Assuming a default policy $\pi^d(s)$ (any policy is available; typically random walk in the case of exploration task), gain function is defined as

$$g(s) = r(s) - \lambda KL\big(\pi(s)|\pi^d(s)\big) \tag{1}$$

where $r(s)$ is expected reward at the state s and $\lambda KL\big(\pi(s)|\pi^d(s)\big)$ is the cost imposed on the difference between the current policy $\pi(s)$ and the default policy $\pi^d(s)$ (λ is a relative weight of the cost). Then, previous works have shown that the optimal policy and corresponding value functions can be determined explicitly by solving linear equations (1–3). Here we consider an environment that consists of N_N nonterminal states and N_T terminal states. We define two transition probability matrices under the default policy: P_{NT} is a $N_N \times N_T$ matrix for transitions from non-terminal states to terminal states, and P_{NN} is a $N_N \times N_N$ matrix for transitions across non-terminal states. Furthermore, $\vec{r}_N$ and $\vec{r}_T$are vectors of rewards at non-terminal states and terminal states, respectively. In this condition, a vector of value functions under optimal policy $\vec{v}^* = \big(v^*(s_1), \dots, v^*(s_{N_N})\big)$ is obtained as

$$\exp(\lambda^{-1}\vec{v}^*) = \mathrm{M}\mathrm{P}_{NT}\exp(\lambda^{-1}\vec{r}_T) \tag{2}$$

where $\mathrm{M} = (\mathrm{diag}(\exp(-\lambda^{-1}\vec{r}_N)) - \mathrm{P}_{NN})^{-1}$ is DR (3).

To relate $\vec{v}^*$ to SI, we consider a specific condition in which the environment consists of non-terminal states, and a virtual terminal state is attached to a goal state s_G arbitrarily chosen from those non-terminal states. When the agent gets to the goal state, it transits to the terminal state with a probability p_{NT}. Furthermore, we assume that rewards at non-terminal states are uniformly negative and reward at the terminal state is positive so that the agent has to take a short path to goal to maximize reward. Specifically, we assume all elements of $\vec{r}_N$ are r_-, and $r_T = r_+(s_G)$ where r_- and $r_+(s_G)$ is arbitrary negative and positive values, respectively. Then, we obtain

$$\exp(\lambda^{-1}\vec{v}^*) = (\mathrm{I} - \exp(\lambda^{-1}r_-)\,\mathrm{P}_{NN})^{-1}\vec{e}(i_G)p_{NT}\exp\big(\lambda^{-1}(r_+(s_G) + r_-)\big) \tag{3}$$

where I is an identity matrix, $\vec{e}(i_G)$ is a standard unit vector in which i_G-th element is 1, i_G is the index of the goal state. Because $0 < \exp(\lambda^{-1}r_-) < 1$, $(\mathrm{I} - \exp(\lambda^{-1}r_-)\,\mathrm{P}_{NN})^{-1}$ is equivalent to a successor representation matrix with a discount factor $\gamma = \exp(\lambda^{-1}r_-)$ (4, 5). Therefore, we obtain

$$\exp\big(\lambda^{-1}v^*(s)\big) = SR^d(s, s_G)p_{NT}\exp\big(\lambda^{-1}(r_+(s_G) + r_-)\big) \tag{4}$$

where $SR^d(s, s_G)$ is SR under the default policy. By setting $r_+(s_G) = -r_- - \lambda\log p_{NT} - \lambda\log P^d(s_G)$ ($P^d(s_G)$ is a probability of visiting the state s_G under the default policy), we finally obtain

$$\lambda^{-1}v^*(s) = \log SR^d(s, s_G) - \log P^d(s_G) = SI^d(s, s_G) \tag{5}$$

where $SI^d(s, s_G)$ is SI under the default policy.

The transition probability matrix for non-terminal states P_{NN} slightly differs between settings with and without a goal because adding a transition to a terminal state changes transition probabilities from the goal state. Therefore, if SI is constructed through exploration of the environment without a goal (as in simulations in this paper), SI slightly deviates from the true value function. Rigorous matching is achieved by infinitesimal p_{NT} (a small transition probability to the terminal state with a large positive reward) in that case.

Details of dimension reduction for DSI vectors. To obtain DSI-decorr, we iteratively updated *D*-dimensional vectors $\vec{x}(s)$ and $\vec{w}(s)$ ($D < N_s$) by Nesterov's accelerated gradient descent method (6) to minimize the following objective function J_{decorr}, rectifying all elements every iteration ($\forall i, x_i(s) \geq 0, w_i(s) \geq 0$).

$$J_{decorr} = \frac{1}{2}\sum_{s,s'} \rho(s,s')\big(PSI(s,s') - \vec{x}(s)\cdot\vec{w}(s')\big)^2 + \frac{1}{2}\beta_{cor}\sum_{i\neq j}\big(Corr(i,j)\big)^2 + \frac{1}{2}\beta_{reg}\sum_{s}(\|\vec{x}(s)\|^2 + \|\vec{w}(s)\|^2) \tag{6}$$

The first term of this objective function is weighted approximation error minimization, the second term works for decorrelation between dimensions, and the third term regularizes representation vectors. In this function, $\rho(s,s')$ is a weight for the square error

$$\rho(s,s') = \frac{1}{N_s V}\left(\frac{1}{M}PSI(s,s') + \rho_{min}\right) \tag{7}$$

where *M* and *V* are mean and variance of PSI, respectively, and ρ_{min} is a small value to avoid zero-weight. $Corr(i,j)$ is a correlation between two dimensions in $\vec{x}(s)$

$$Corr(i,j) = \frac{\sum_s \tilde{x}_i(s)\tilde{x}_j(s)}{\sqrt{\sum_s\big(\tilde{x}_i(s)\big)^2}\sqrt{\sum_s\big(\tilde{x}_j(s)\big)^2}} \tag{8}$$

where $\tilde{x}_i(s) = x_i(s) - \frac{1}{N_s}\sum_s x_i(s)$.

Gradients of the objective functions for iterative updates are

$$\frac{\partial J_{decorr}}{\partial x_k(s)} = -\sum_{s'}\rho(s,s')\big(PSI(s,s') - \vec{x}(s)\cdot\vec{w}(s')\big)\,w_k(s') + \beta_{cor}\sum_{j\neq k}\frac{Corr(k,j)\tilde{x}_j(s)}{\sqrt{\sum_s\big(\tilde{x}_k(s)\big)^2}\sqrt{\sum_s\big(\tilde{x}_k(s)\big)^2}} + \beta_{reg}x_k(s) \tag{9}$$

$$\frac{\partial J_{decorr}}{\partial w_k(s')} = -\sum_{s'}\rho(s,s')\big(PSI(s,s') - \vec{x}(s)\cdot\vec{w}(s')\big)\,x_k(s) + \beta_{reg}w_k(s') \tag{10}$$

We note that we regarded mean and variance of $x_k(s)$ in the correlation ($\sum_s(\tilde{x}_k(s))^2, \sum_s(\tilde{x}_k(s))^2$ in Eq. (8)) as constants in the calculation of these gradients. Practically, this heuristic did not affect the performance of decorrelation.

To obtain DSI-sparse, we performed the same procedure with DSI-decorr but we changed the objective function to

$$J = \frac{1}{2}\sum_{s,s'} \rho(s,s')\big(PSI(s,s') - \vec{x}(s) \cdot \vec{w}(s')\big)^2 + \beta_{sparse}\sum_{s,k}\big(x_k(s) + w_k(s)\big) \quad (11)$$

We note that the second term of this function is equivalent to a L-1 constraint because $x_k(s)$ and $w_k(s)$ are non-negative. Gradients are

$$\frac{\partial J_{sparse}}{\partial x_k(s)} = -\sum_{s'} \rho(s,s')\big(PSI(s,s') - \vec{x}(s) \cdot \vec{w}(s')\big)\, w_k(s') + \beta_{sparse} \quad (12)$$

$$\frac{\partial J_{sparse}}{\partial w_k(s')} = -\sum_{s'} \rho(s,s')\big(PSI(s,s') - \vec{x}(s) \cdot \vec{w}(s')\big)\, x_k(s) + \beta_{sparse} \quad (13)$$

Throughout this paper, the learning rate was 0.05 and the number of iteration was 10000. Parameters were β_{cor}=1, $\beta_{reg} = 0.001$, β_{sparse}=0.0001, and $\rho_{min} = 0.001$.

Calculation of SR. Throughout this study, we used a direct count method because we performed only offline processing of finite data. In a sequence of states $\{s_1, \dots, s_t, \dots, s_T\}$, we recursively calculated exponential traces of past states $z(s,t) = \sum_{\tau=0}^{t-1} \gamma^\tau \delta(s_{t-\tau}, s)$ as

$$z(s,t) = \gamma z(s,t-1) + \delta(s_t, s) \quad (14)$$

where $\delta(i,j)$ is Kronecker's delta and γ is a discount factor. We calculated SR from state counts and coincidence counts as

$$SR(s,s') = \frac{\sum_{t=1}^{T} z(s,t)\, \delta(s_t, s')}{\sum_{t=1}^{T} \delta(s_t, s)} \quad (15)$$

Learning DSI in 2-D spaces. As an environment, we assumed a square room (or interconnected separated rooms) tiled with 30 × 30 discrete states. In each simulation trial, an agent starts at one of those 900 states and transits to one of eight surrounding states each time except that transitions are limited at states along the boundary (the structure was not a torus). Transitions to surrounding states occur with an equal probability. We performed 500 simulation trials and obtained a sequence of 100,000 time steps in each trial. We calculated occurrence probabilities ($P(s)$) and a successor representation matrix ($SR(s,s')$) of 900 states from those sequences, and calculated PSI and DSI (100-dimensional) as described in the Model section. The discount factor γ was set to 0.99.

We repeated this procedure five times using different random seeds which gave different representations each time, to report reproducibility or statistics of results.

We also generated structures of the mazes shown in Fig. S15 by "random_maze()" function of mazelab (7) and calculated DSI in the same way. For mazes, dimensions of compressed DSI

representation vectors were changed to 50 because the number of nodes were smaller than other spatial environments and dimension reduction became unstable.

Gridness analysis. In analyses of spatial representations, we classified grid and non-grid cells based on gridness scores following the procedure in previous studies (8–11). For each unit, we first determined the radius of a central peak in the spatial autocorrelation map following the criterion in previous study (9). We defined a circular area with outer radius R excluding the central peak (an annulus), centered on the origin of the spatial autocorrelation map. We calculated correlations between the original and rotated maps in the annulus. Gridness was defined as the difference between the lowest correlation at 60° and 120° and the highest correlation at 30°, 90° and 150°. We repeated this calculation incrementing R from R_{min} to R_{max}, and we determined gridness of the unit as a maximum value across all settings of R. For a simple 2-D room (the size was 30 x 30), $R_{min} = 8$ and $R_{max} = 18$. For "the context Φ" in spatial inference task (the size was 21 x 21), $R_{min} = 6$ and $R_{max} = 14$.

A unit was immediately classified as a non-grid cell when gridness did not exceed 0.3. For candidate grid cells (gridness>0.3), we performed a field-shuffling analysis (10, 11) to confirm that gridness is not obtained by chance. Briefly, we segmented spatial fields in a spatial representation map using a watershedding algorithm. We generated a shuffled map by randomly replacing each field to another position. We first replaced a peak bin, then bins around the peak were incrementally replaced, keeping the relative position to the peak bin. When the target bin had already been occupied by other fields, the nearest empty bin was used as the target bin instead. We created 100 shuffled maps and calculated gridness of shuffled maps to construct a null distribution. The unit was judged as a grid cell if gridness exceeds the 95 percentile of the null distribution.

Path integration by DSI vectors. In the path integration task, we performed the estimation of states at each time step s_t from an initial state s_0 and a sequence of movements $\{a_0, a_1, \cdots, a_{T-1}\}$ (a_t represents one of eight directional movements). To perform path integration, we initialized $\vec{z}_0$ by a DSI representation vector $\vec{x}(s_0)$, then we made an estimate of the next representation vector $\vec{z}_{t+1}$ by linear transformation of the current representation vector $\vec{z}_t$ as

$$\vec{z}_{t+1} = \mathrm{M}(a_t)\vec{z}_t \tag{16}$$

where $\mathrm{M}(a_t)$ is movement-conditional recurrent weight matrix. We determined a position at each time step by searching a DSI representation vector $\vec{x}(s)$ that has the minimum Euclidian distance with the estimated vector as

$$s_t = \mathrm{argmin}_s \|\vec{x}(s) - \vec{z}_t\|_2 \tag{17}$$

Before the estimation, we optimized the matrix $\mathrm{M}(a_t)$ by minimizing prediction error $\|\vec{x}(s_{t+1}) - \mathrm{M}(a_t)\vec{x}(s_t)\|_2^2$ by stochastic gradient descent during random walk in the environment (20 simulation trials of 100,000 time steps).

Goal-directed spatial navigation by DSI vectors. In each trial, we sampled a start location (state s_{init}) and a goal location (state s_G) such that the shortest path length is minimally 10, and an agent has to navigate between them. The rule of the state transition for navigation was as follows: suppose that the agent exists at a state s, and a set of neighboring states of s is $A(s)$. Given the goal representation vector $w(s_G)$, a value of a neighboring state $s_{next} \in A(s)$ is estimated by $x(s_{next}) \cdot w(s_G)$, and the agents transits to the state that has a maximum value. Because of the relationship between value functions and DSI, this rule approximate value-based decision making for goal-directed navigation. However, because of approximation, the agent sometimes deviated from optimal paths. Specifically, the agent was sometimes trapped in local loops of a few adjacent states, which significantly impaired the performance. To avoid that, we heuristically introduced a "familiarity" variable $f(s)$ to each state. This variable was initially set to zero and incremented by one each time the agent visits the state s. Then, value was evaluated by $x(s_{next}) \cdot w(s_G)$- $f(s_{next})$, which facilitates escape from local loops. Note that this strategy alone was not enough for appropriate goal-directed navigation (see results in Fig. 6 for example).

In the evaluation, we terminated the simulation when the agent did not reach the goal in 100 time steps, and we considered the path length was 100 in the terminated trial.

Learning DSI from text data. We used text data taken from English Wikipedia dump (enwiki-latest-pages-articles, 22-May-2020). We first generated text files from raw data using wikiextrator (https://github.com/attardi/wikiextractor). We tokenized texts by nltk Punkt sentence tokenizer, and randomly sampled 100,000 articles containing 1,000 tokens at minimum. We lowercased all characters and removed punctuation characters in the data. After that, we selected words that appeared more than 1,000 times in the data and substituted all other rare words by <unk> symbol. Finally, we obtained data that contains 124M tokens and 9376 words (i.e. 9376 states).

The procedure of calculating DSI was same as the case of 2-D spaces except that the discount factor γ was set to 0.9. The setting of other parameters was the same as the experiment of 2-D spaces. Using the preprocessed data, we repeated learning of DSI vectors five times using different random seeds (i.e. different initial values for optimization) which gave different representations each time, to report reproducibility or statistics of results.

We also performed same tokenization, word selection, lowercasing and punctuation removal to training dataset (wiki.train.tokens) in WikiText-103 word level dataset (12) for

comparison in Fig. S8. Sampling of articles was not performed. Finally, we obtained text dataset containing 87M tokens and 7517 words.

Comparison with other word embedding methods. We compared DSI with other word embedding models applied to the same text data under same dimensionality (300 dimensions). For skip-gram and continuous-bag-of-words (CBOW) (13, 14), we used implementation in Python Gensim library. For GLoVe (15), we used implementation by Pennington et al. (https://github.com/stanfordnlp/GLoVe). PPMI-SVD (16) and SR-SVD (5) were implemented by ourselves. The window size of coincidence count was set to 10 in all models, and other parameters followed default settings or parameters described in original papers. The discount rate of SR-SVD were same as SR used for calculation of DSI ($\gamma = 0.9$). For BERT, we took representations in a pretrained model (bert-base-uncased in Hugging Face transformers) (17, 18), thus training data and dimensionality (768 dimensions) were different from our setting.

Evaluation of the semantic structure of DSI vectors. To analyze the semantic structure in representation vectors, we considered ten semantic categories (“mammal”, “building”, “vehicle”, “food”, “clothing”, “body part”, “computer”, “feeling”, “creator”, “relative”) and chose ten words for each category as follows.
“mammal”: dog, cat, bull, bear, elephant, fox, horse, lion, rat, tiger
“building”: building, church, theater, hall, school, hotel, house, library, mansion, palace
“vehicle”: vehicle, ambulance, bike, aircraft, bus, car, helicopter, locomotive, rocket, ship
“food”: food, bread, cheese, candy, rice, beer, coffee, milk, tea, wine
“clothing”: clothing, shirt, hat, belt, costume, dress, wear, cap, coat, crown
“body part”: arm, bone, lung, ear, eye, finger, foot, hair, kidney, leg
“computer”: computer, software, internet, code, access, server, website, pc, node, portal
“feeling”: feeling, anger, anxiety, comfort, confusion, emotion, enthusiasm, happiness, joy, love
“creator”: creator, architect, artist, composer, designer, producer, filmmaker, photographer, musician, painter
“relative”: husband, brother, aunt, cousin, daughter, father, grandfather, grandmother, mother, sister
We took DSI representation vectors for these 100 words and computed dissimilarity (1-Pearson’s correlation coefficient). MDS was performed using the same dissimilarity metric.

Analogical inference of words. In Mikolov’s dataset, many sets of four words are given. There is a relationship “WORD1 is to WORD2 as WORD3 is to WORD4” (i.e. king is to queen as brother is to sister). Then, an expected relationship in the vector space is WORD2-WORD1=WORD4-WORD3. In this study, we performed inference of WORD4 by WORD3+WORD2-WORD1. We regarded an inference was correct if the actual vector of

WORD4 had the largest cosine similarity to the inferred vector among all word representation vectors (except those for WORD1, WORD2, and WORD3). If the number of words is 10,000, a chance level of the correct answer rate is 0.01%. Therefore, the performance shown in this study (more than 50%) is far above the chance level.

When we performed analogical inference by calculation of the limited number of dimensions (partial recombination), we first calculated $\vec{x}(WORD2) - \vec{x}(WORD1)$ and then identified the n largest and the n smallest elements in that vector. We summed those $2n$ out of 300 elements to $\vec{x}(WORD3)$ (in other words, other elements of $\vec{x}(WORD2) - \vec{x}(WORD1)$ were substituted by zero before summation). Correctness of the inference was evaluated by the same way with the original task.

Quantitative evaluation of conceptual specificity. Conceptual specificity of each unit was evaluated using WordNet database (19). In WordNet, a word belongs to several synsets (sets of cognitive synonyms), and semantic similarity of two synsets can be evaluated from the shortest path length between them in the WordNet structure (we used path similarity function in nltk library). We defined similarity of two words as the highest similarity among all combinations of synsets of those words. We calculated mean similarity of all combinations of TOP-10 words (ten words that highly activated the unit; Fig. 3A) that are available in WordNet. We evaluated only units which had at least five TOP-10 words available in WordNet. Furthermore, we randomly generated 1,000 pairs of words available in WordNet, and generated a null distribution of similarity between words. We defined a significance threshold of similarity as a 95 percentile of the null distribution, and a unit was classified as a significantly concept-specific unit if mean similarity of TOP-10 words exceeded the threshold. Furthermore, we quantitatively defined a conceptual specificity of each unit as

$$\frac{s_{unit}}{s_{null}} - 1 \tag{18}$$

where s_{unit} is mean similarity of TOP-10 words and s_{null} is the mean of the null distribution. This quantity becomes zero if similarity between TOP-10 words is not different from random pairs, and becomes positive if TOP-10 words are semantically similar. This conceptual specificity was averaged over all evaluated units.

Of note, we found several non-significant units exhibit conceptual specificity according to manual inspection (see SI Appendix, Fig. S5). This is probably because of the limitation of knowledge covered by WordNet. Therefore, we suppose that the current evaluation method tends to underestimate the number of concept-specific units. However, the comparison across models was fair because we used the same procedure and criteria for all models.

Analogical inference of spatial contexts. We considered a 2-D space tiled with 21x21 (441) states and four spatial contexts (barrier layouts) A, B, Φ, A+B (SI Appendix, Fig. S11). We

define separated states in contexts A, B, Φ, A+B as s_i^A, s_i^B, s_i^Φ and s_i^{A+B} where i is a positional index which indicates a same position in all contexts ($i = 1,2,\ldots,441$). We constructed representation vectors for contexts A, B, Φ through direct experiences, then we created $\vec{x}(s_i^{A+B})$ and $\vec{w}(s_i^{A+B})$ as

$$\vec{x}(s_i^{A+B}) = \vec{x}(s_i^A) + \vec{x}(s_i^B) - \vec{x}(s_i^\Phi) \tag{19}$$

$$\vec{w}(s_i^{A+B}) = \vec{w}(s_i^A) + \vec{w}(s_i^B) - \vec{w}(s_i^\Phi) \tag{20}$$

We performed spatial navigation in a given context using one of four representations $\{\vec{x}(s_i^A), \vec{w}(s_i^A)\}$, $\{\vec{x}(s_i^B), \vec{w}(s_i^B)\}$, $\{\vec{x}(s_i^\Phi), \vec{w}(s_i^\Phi)\}$, and $\{\vec{x}(s_i^{A+B}), \vec{w}(s_i^{A+B})\}$ for corresponding positions, following the vector-based decision making rule described in the previous section ("Goal-directed spatial navigation by DSI vectors").

To learn representations in contexts A, B, and Φ, we sampled sequences of $\{s_i^A\}_{i=1,\cdots,441}$, $\{s_i^B\}_{i=1,\cdots,441}$, and $\{s_i^\Phi\}_{i=1,\cdots,441}$ as in the previous section ("Learning DSI in 2-D spaces"). However, we performed random walk in the 2-D space while switching between context A, B, and Φ, that is, state transitions to the same position in other contexts occurred every 5,000 time steps (transition between s_i^A, s_i^B, and s_i^Φ). It means that we assumed that the setting of barriers can change during the experience. This transition was introduced to associate the same position in different contexts. We performed 500 simulation trials and obtained a sequence of 100,000 time steps in each trial. From sampled sequences, we calculated SR for all combinations of $\{s_i^A\}_{i=1,\cdots,441}$, $\{s_i^B\}_{i=1,\cdots,441}$, and $\{s_i^\Phi\}_{i=1,\cdots,441}$ and calculated 100-dimensional DSI vectors for 1,324 (441×3) states by simultaneous compression of all states (dimension reduction of a 1,324×1,324 PSI matrix). The discount factor γ was set to 0.99.

When we performed the inference by calculation of the limited number of dimensions, we first identified the n units (dimensions) that had the largest representational distances between the context B and Φ. The representational distance of the unit k (k-th dimension of vectors $x_k(s)$) was defined as

$$\sum_i \left(x_k(s_i^B) - x_k(s_i^\Phi)\right)^2 \tag{21}$$

We calculated $x_k(s_i^{A+B}) = x_k(s_i^A) + x_k(s_i^B) - x_k(s_i^\Phi)$ only for those n dimensions, and we set $x_k(s_i^{A+B}) = x_k(s_i^A)$ in other dimensions.

Statistical analysis. To ensure reproducibility, we showed results obtained by five repetitions of simulations with different random seeds (different initial values for learning and simulations). We applied two-sided t-tests to those five samples assuming normality of data, and detailed values of t-statistic and p-values are summarized in Table S1, S2, and S3. Significance thresholds were modified by Bonferroni correction.

Extended Discussion

Relationships with disentangled visual representation learning. In our dimension reduction method, we introduced non-negativity, sparsity, decorrelation and regularization as constraints. Those constraints are also important for extraction of independent components (20–22), and it is known that imposing independence in latent spaces of deep generative models results in the emergence of disentangled representations for visual features (22, 23). As those disentangled visual representations explain single-cell activities in higher-order visual cortex (23), we similarly interpreted disentangled word representations in our model as concept cells. Concept cells respond to a specific concept, whereas population-level activity patterns represent abstract semantic structures (24). Such property is consistent with the factorized and distributed nature of disentangled representation vectors. Our model provides the view that concept cells emerge as disentangled representations for semantic concepts.

Prediction for concept cells in the population level. The biological interpretation of our model predicts a population-level property of concept cells. When a human subject (or an animal) learns a novel word (or an item) that can be interpreted by the composition of previously learned concepts, the representation for the novel item can be created by partial recombination of previous formed concept cells, instead of creating novel concept cells. Concept cells exhibit concept-specific activities, but they can simultaneously work as building blocks to represent specific items as assemblies (24, 25). Thus, recombination of those assemblies is useful for creation (or prediction) of novel conceptual representations. This strategy is beneficial in term of energy efficiency: the brain can reduce the number of neurons for processing by using factorized representations, and switching only a part of assemblies requires a smaller number of synapses and plastic changes than overall control of the neural population. Our model confirmed that such efficient computational strategy actually works for semantic concepts learned from the text data in the real world.

Possible functional difference between EC and HPC. In the DSI model, different constraints in optimization (decorrelation or sparsity) switch the formation of grid-like (EC) and place-specific (HPC) representations. In contrast, switching constraints created no qualitative differences in semantic representations. Consistently with our results, similar concept cells exist in both EC and HPC in the human brain (24, 25). However, the sparseness constraint suppresses neural activity to impose low energy consumption, whereas the decorrelation constraint orthogonalizes coding vectors to enhance information capacity. Therefore, they

benefit brain computing differently, and functional differences may be revealed by further assessments of representations. Furthermore, our model did not account for various representational forms in EC such as head direction cells (8) and object vector cells (26). An improved model or a simulation setting that generates the missing representations is necessary to clarify the dissimilarity between EC and HPC. What semantic information such studies may add to neuronal representations in EC is an intriguing open question. Furthermore, HPC and EC may differ in the learning speed. BTSP rapidly organize novel place cells in hippocampus (27) but grid cells are relatively stable across environments. Therefore, HPC may represent specific concepts for recent episodes, whereas EC represents a robust conceptual framework obtained through long-term experiences.

Furthermore, DSI-decorr could decompose context-dependent information as non-grid representations (Figure 6). This suggests that EC may independently encode reusable information of the 2-D space (as grid cells) and context-specific information of non-uniform spatial structures (as non-grid cells). More generally, it may be efficient for EC to decompose context-dependent and independent structures of tasks and concepts. Such a generalization is to be investigated in future study.

Roles of constraints in DSI. In DSI, we mainly used three constraints for learning: non-negativity, decorrelation and sparsity. The need for sparsity is relatively clear because place cells and concept cells seem sparse and local representations. Below, we discuss why non-negativity and decorrelation are necessary in our model.

Non-negativity is important for both grid-like representations and conceptual representations (22, 28–31). How hexagonal grids emerge under non-negativity was thoroughly discussed in the previous work (30), but the effect of non-negativity in word vector learning is underexplored. Intuitionally, word vector learning maximizes the inner products of vectors for related words. If all elements are non-negative, the number of non-zero elements shared between two vectors roughly determines the inner product between them. Thus, each non-zero element should correspond to a shared factor between two words, and a semantic factor in each dimension emerges for a group of semantically related words. This intuitively accounts for the role of non-negativity although it should be further investigated in future research.

Decorrelation in our model imposes approximate orthogonality, which previously was used to generate grid-like representations (28). As grid patterns are orthogonal eigenvectors of SR in 2-D spaces (5), we can expect that the appropriately orthogonalized dimensional reduction of SR yields some kinds of grid patterns with various phases and scales. Furthermore, grids are forced to be hexagonal, neither square nor stripe, under the non-negativity constraint (30). However, neural network models optimized for path integration can generate grid-like representations without decorrelation constraints (30). This suggests that we may substitute the decorrelation constraint in our model with other constraints or functional demands. We

speculate that “Mexican-hat” shapes of place fields play an important role in the previous studies (28, 30). In contrast, SR in 2-D spaces does not take Mexican-hat shapes, leading to different constraints between DSI and the other models.

Biological plausibility of the timescale of prediction in the model. In this section, we quantitatively discuss biological interpretations of γ. Specifically, we argue that the setting of γ in this work falls into a biologically plausible range under the assumption that SR-like neural representations are created by behavioral time scale synaptic plasticity (BTSP) (82), and a gradient of representational timescales (83) exists in hippocampus. We note that our interpretation depends on rough estimates based on experimental data from rodents and humans, and we consider both spatial and temporal scales of learning that are linked by the speed of animal’s locomotion and human’s reading.

First, we discuss the timescale of word representation learning. A candidate mechanism for the emergence of SR-like representations in hippocampus is behavioral time scale synaptic plasticity (BTSP) in CA3-to-CA1 synapses (27). Therefore, we consider the timescale of BTSP for estimating a biological plausible value of γ. BTSP, which is triggered by calcium plateau (large bursting events due to calcium influx) of CA1 neurons, potentiates synapses even when activations of presynaptic and postsynaptic neurons are temporally distant, unlike classical spike-timing-dependent plasticity in the hippocampus. Furthermore, the timescale of synaptic potentiation is longer in the backward direction (pre-post order) than in the forward direction (post-pre order). Therefore, BTSP causes one-shot formation of predictive place fields like SR (27, 32). Exponential fits of the amplitude of synaptic potentiation yielded time constants $\tau_b = 1.31s$ in backward and $\tau_f = 0.69s$ in forward in the mouse hippocampus (27).

Suppose that BTSP occurs in the hippocampus of a human listening or reading a language. The reading rate of human adults falls in the range of 3 – 5 words per second (180 to 300 words per minute) (33). Assuming that the time constant of BTSP is 1 - 2 s, we can estimate a time constant of BTSP for words roughly as τ=3 – 10 words. Our model succeeded in word representation learning when we used $\gamma = 0.8$ and $\gamma = 0.9$ (SI Appendix, Fig. S8), which correspond to $\tau \approx 4.5$ words and $\tau \approx 9.5$ words, respectively (noting that $\gamma = \exp(-\tau^{-1})$). Therefore, the setting of γ for word representation learning falls into a biologically possible range.

In contrast, successful spatial navigation and path integration needed relatively large discount rates ($\gamma \geq 0.99$). The lower bound $\gamma = 0.99$ corresponds to $\tau \approx 100$, which is ten times larger than the time constant for word representation learning. If we assume that the time constant $\gamma = 0.9$ ($\tau \approx 9.5$) corresponds to BTSP (1 – 2 s), $\gamma = 0.99$ ($\tau \approx 100$) requires the time constant of 10 – 20 seconds. Furthermore, if we assume that the 30 x 30 space (Fig. 1) corresponds to a 150 cm x 150 cm square box for a grid-cell experiment (8), a rat’s motion at each timestep is approximately 5 cm, so the timescale $\tau \approx 100$ indicates the spatial scale

5 m. This is much longer than the place field size formed by BTSP (approximately 130 cm on a linear track (27)), suggesting that the scale of experimentally observed BTSP is not enough for spatial learning of DSI. This is natural from the view of reinforcement learning because γ determines the temporal range of prediction of future rewards: long-range navigation to a distant goal requires a large discount rate for prediction in large temporal and spatial scales.

Such long temporal and spatial scales for learning is biologically plausible because there is a gradient of timescales of hippocampal representations along the longitudinal axis (34–38). In the rodent hippocampus, it has been reported that the width of place fields in the ventral hippocampus exceeds ~10 m, which is ten times larger than that in the dorsal hippocampus (~1 m; corresponding to the scale of experimentally observed BTSP) (35), and a similar difference in the representational scales has been found in the human hippocampus along the anteroposterior axis (36). Therefore, a natural interpretation is that the anterior (ventral for rodents) hippocampus provides the long timescale for long-range spatial navigation, whereas the posterior (dorsal for rodents) hippocampus provides the short timescale for word representation learning. With this interpretation, the spatial scale 5 m (corresponding to $\tau \approx 100$) in our model falls into a biologically plausible range (< 10 m). This view is also supported by several experiments suggesting that the human anterior (rodent ventral) hippocampus engages in navigation in the complex spatial structures and representations of distant goals (39–42).

Learning SR in various timescales is useful for flexible decision making and planning in various scales (38). While large discount rates support long-range navigation, small discount rates are useful for decision making in short timescales (e.g. short-range spatial navigation and immediate choices after cue presentations). Furthermore, learning semantic representations in a long timescale may enable the extraction of global and coarse-grained semantic information (gist) rather than details (the meaning of each word) (37). Building multiscale DSI model for more flexible computations is an intriguing future research direction.

Furthermore, navigation in longer scales may depend on hierarchical abstraction of states and actions, and setting subgoals between a start and a goal, as in hierarchical reinforcement learning (43, 44).

Possible biological implementations of DSI. Although DSI has a clear mathematical interpretation, how biological neural networks can learn DSI is unclear. A possible solution is an extension of Skip-gram neural network (13, 14) with SR, nonnegativity, and decorrelation (or sparsity). Because SI corresponds to pointwise mutual information (PMI), which is the optimum of Skip-gram neural network (16), we can expect that an extended Skip-gram network learns DSI. The learning algorithm of skip-gram consists of the prediction (association) of temporally close words in data and random sampling of words that induces negative learning balancing against positive association (negative sampling). By interpreting these two

components as BTSP (27) and hippocampal replay (45), respectively, we can speculate that hippocampal learning in awake-sleep cycles corresponds to DSI learning. Building such a model and exploring the underlying circuit mechanisms in the hippocampus and EC are open for future research.

Alternative biological interpretations of DSI. We interpreted conceptual units in our model as concept cells in HPC and EC because our model also produces place cells and grid cells. However, we can also interpret these units as “semantic features” (attributes) in a broad sense (46–50). A previous study has shown that distributed word representations composed of combinations of semantic features are useful for decoding words from fMRI activity patterns (49). It has also been shown that skip-gram representations support high-performance decoding of semantic information from fMRI data (51). In those experiments, semantic features are associated to brain-wide activity patterns, which implies that conceptual units in our model may also be related to brain areas other than HPC and EC. Especially, our model can be related to computations in two brain regions: anterior temporal lobe (ATL) and prefrontal cortex (PFC). It has been proposed that ATL processes semantic cognition and memory through interactions with multimodal sensory features across brain areas (46, 50). DSI model may be related to the computational mechanism of semantic features in ATL. As for PFC, fMRI studies have shown hexagonal modulations of neural activities in 2-D conceptual spaces as in EC (52–54). Furthermore, contributions of PFC to spatial navigation (55) and verbal analogical reasoning (56) have been suggested. Although concept-specific neural representations have not been found in PFC, other properties of DSI are consistent with those of PFC. These other possibilities should be investigated in future studies.

The role of HPC and EC in spatial tasks. The contribution of grid cells in EC to path integration has been suggested by experiments with rats and mice (57, 58). However, there is a report that human patients with lesions of HPC and EC could normally perform path integration when the task depends only on working memory (59). Therefore, it is possible that other brain areas, such as parietal cortex (60) and retrosplenial cortex (61), also contribute to spatial tasks in the human brain. This point should be carefully validated experimentally and computationally in the future, appropriately dissociating contributions of memory and spatial functions.

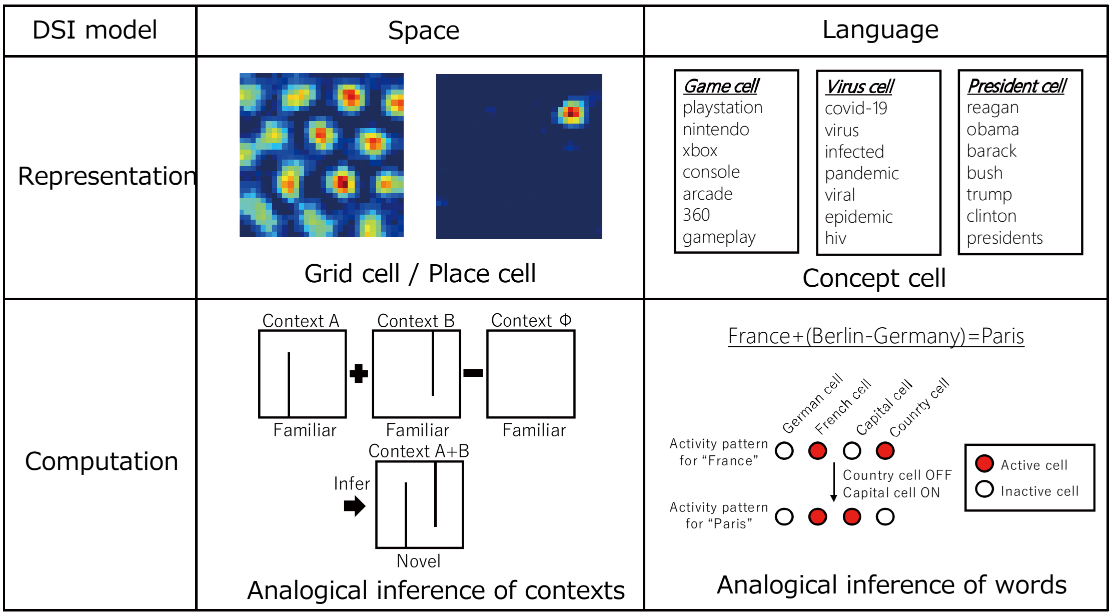

Fig. S1. Summary of DSI model. DSI creates spatial representations like grid cells or place cells, whereas DSI also creates concept-specific word representations like concept cells. Furthermore, DSI offers a common computational framework for spatial and semantic inferences based on analogical relationships.

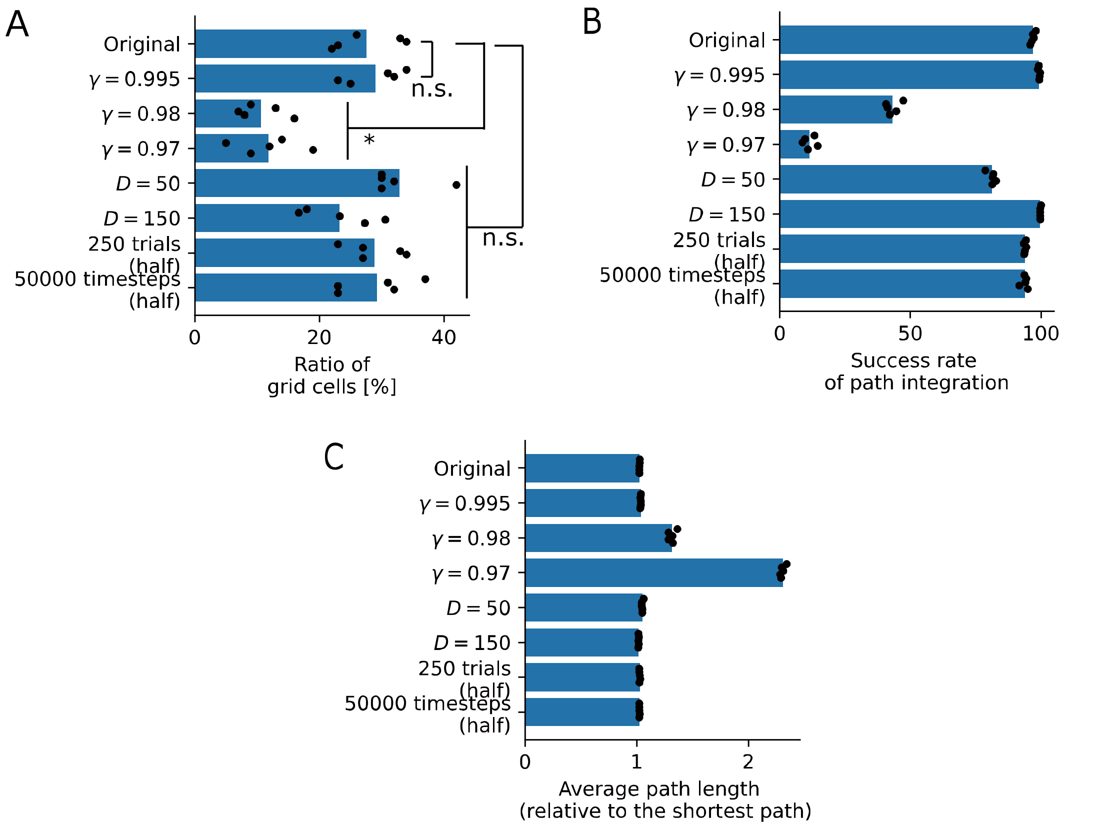

Fig. S2. Evaluation of spatial representations obtained by DSI-decorr with various parameter settings ($\gamma = 0.97, 0.98, 0.995$, $D = 50, 150$, 250 simulation trials for learning, 50000 timesteps in each simulation trial). The original setting shown in the main figure was $\gamma = 0.99$, $D = 100$, 500 simulation trials for learning, 100000 timesteps in each simulation trial, and only one parameter was altered from the original setting in each test. Black dots indicate five repetitions with different random seeds, and bars indicate mean values in each setting. (A) Ratio of grid cells. (B) Success rate of path integration (evaluated by 1000 randomly sampled paths with 10 transitions). (C) Average path lengths in the spatial navigation task (same as Figure 2, S3, S4). *P<0.05; n.s., not significant. All statistical tests were two-sided t-tests and significance thresholds were modified by Bonferroni correction.

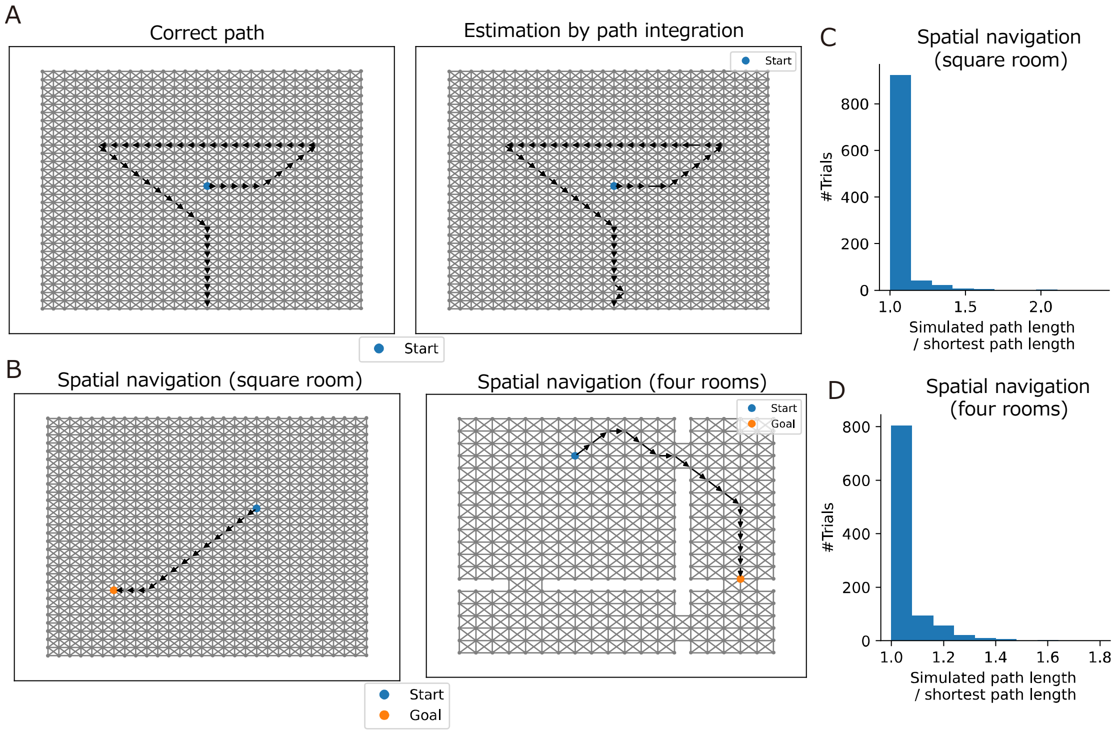

Fig. S3. Spatial navigation using DSI representation vectors (DSI-sparse). (A) Path integration using DSI model. (Left) Actual path. (Right) Path estimated from DSI vectors updated by movement information. (B) Example spatial paths obtained by DSI-based navigation. (C) A histogram of path lengths in 1,000 trials of spatial navigation in the square-room environment. Note that a start and a goal were randomly determined in each trial, and we normalized a simulated path length by the shortest path length between the start and the goal. (D) A histogram of path lengths in the four-room environment.

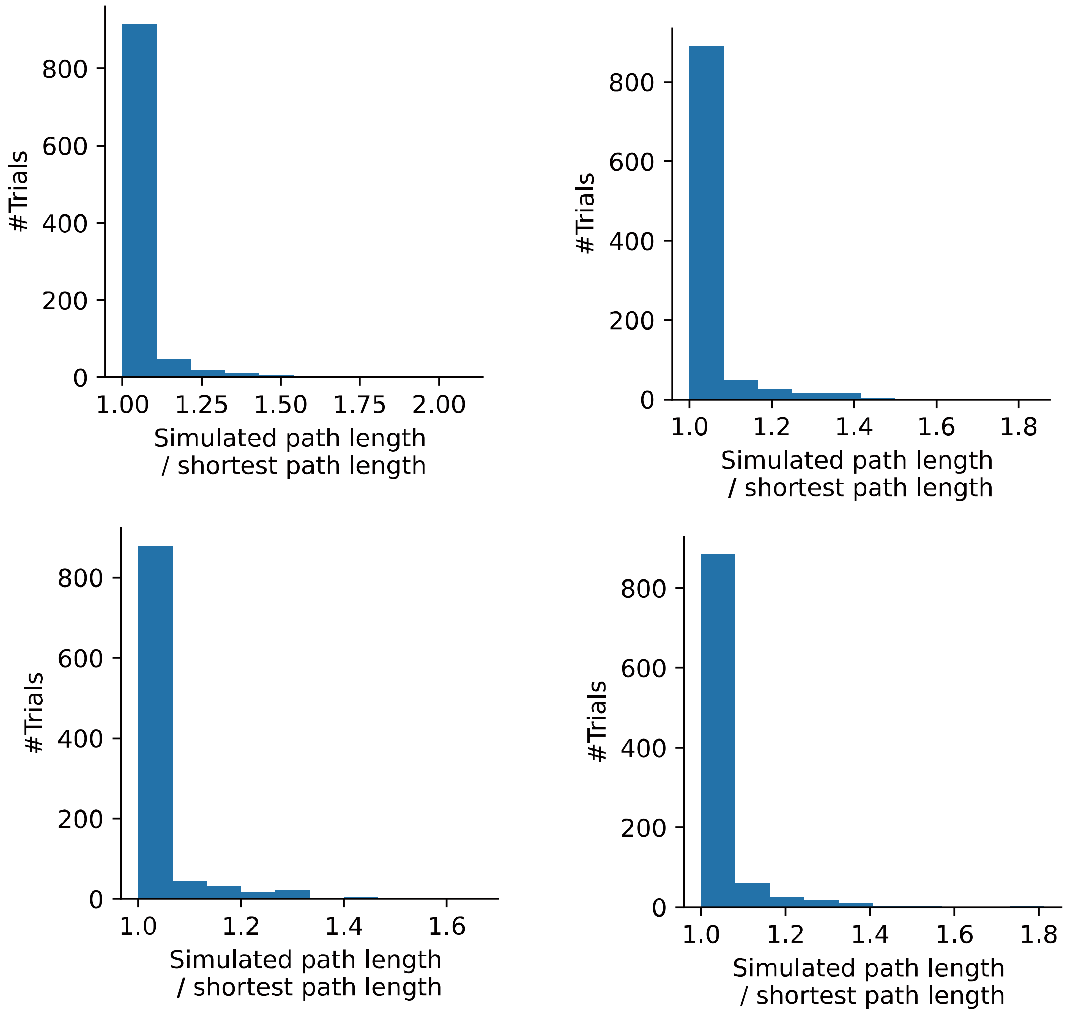

Fig. S4. Histograms of path lengths in 1,000 trials of spatial navigation in the square-room environment obtained by 4 simulations with different random seeds. Note that a start and a goal were randomly determined in each trial, and we normalized a simulated path length by the shortest path length between the start and the goal.

Unit 1	Unit 2	Unit 3	Unit 4	Unit 5
faced	transit	humid	universities	sheets
overcome	commuter	winters	colleges	olivia
facing	trains	summers	uc	compact
due	bus	climate	usc	senators
owing	buses	mild	consortium	antony
citing	subway	precipitation	institutes	proposes
experiencing	rail	warm	carnegie	atlas
cope	passenger	temperatures	affiliated	spaces
amid	metro	cool	alumni	geometry
suffered	passengers	cold	selective	rough

Unit 6	Unit 7	Unit 8	Unit 9	Unit 10
designers	rebounds	nbc	eleventh	nhl
scientists	assists	aired	tenth	confirmation
experts	averaged	abc	ninth	hockey
professionals	steals	airing	seventh	maple
consumers	nba	cbs	eighth	roller
researchers	averaging	syndicated	twelfth	judiciary
filmmakers	partition	programming	7th	flames
lawyers	points	broadcast	8th	devils
composers	prussia	channel	10th	batch
scholars	mvp	espn	11th	senators

Unit 11	Unit 12	Unit 13	Unit 14	Unit 15
lowest	valve	archive	hatch	rotten
ranked	wheel	archives	deaf	tomatoes
highest	steering	manuscripts	robot	metacritic
ranks	rotating	collections	suffrage	aggregate
literacy	wheels	collection	egg	approval
demographic	cylinder	manuscript	seymour	reviews
rising	trigger	library	salem	grossed
prevalence	shaft	catalogue	wwe	rating
ratings	pump	valuable	monte	byron
rank	gear	documents	resist	apparatus

Fig. S5. A part of word representations formed by DSI-decorr. Ten words that gave the highest activation (TOP-10 words) are shown for units 1 - 15. Those classified as concept-specific units in our analysis are boxed with bold lines.

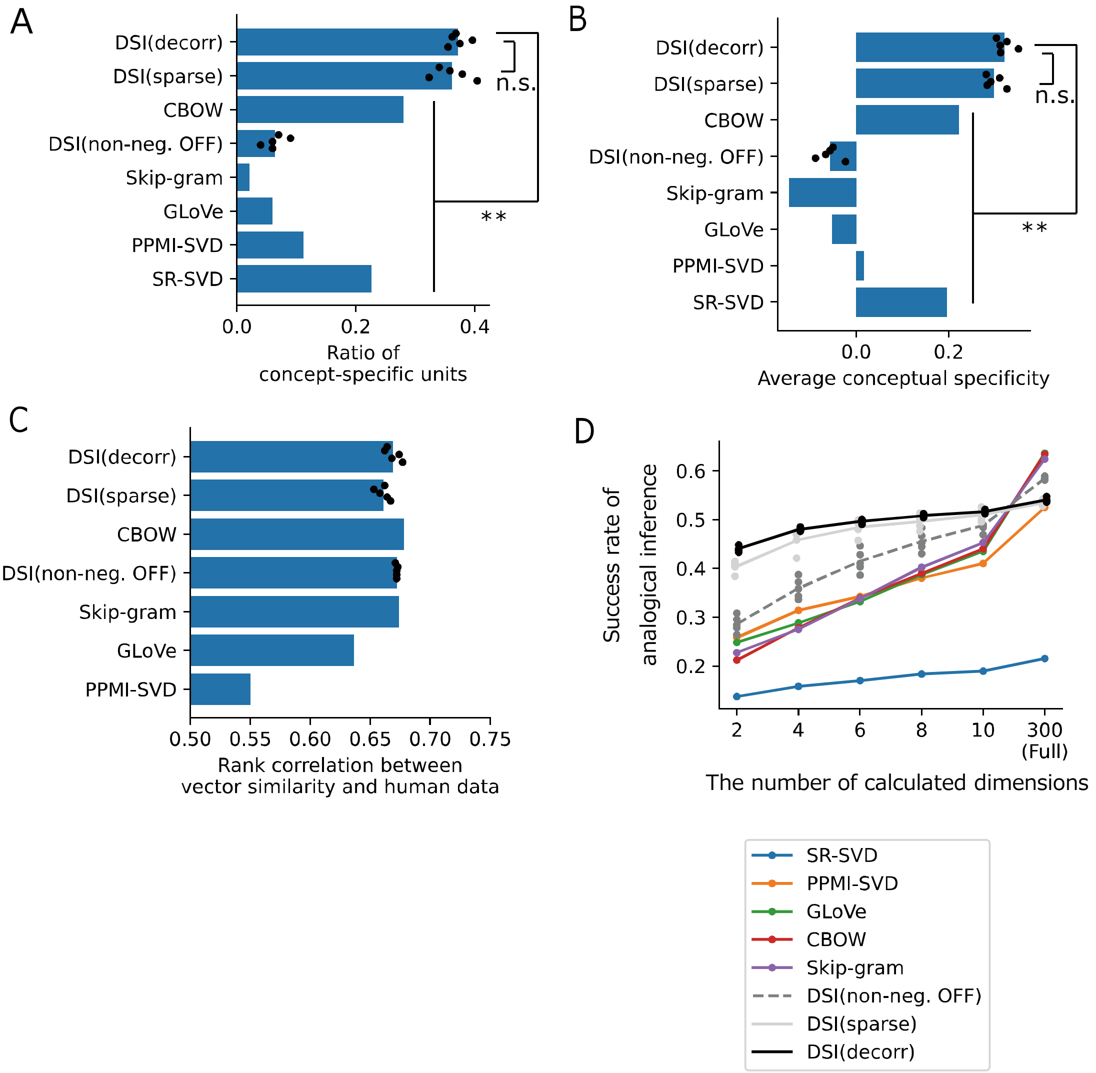

Fig. S6. Evaluation of word representations obtained by DSI and other word embedding methods (D=100). For DSI, dots indicate 5 trials with different random seeds (different initial values for learning); bars and lines indicate means of those 5 simulations. (A) Ratio of concept-specific units. (B) Average conceptual specificity. (C) The rank correlation of word similarity evaluated by word representation vectors (cosine similarity) and humans (WS353 dataset). (D) Success rates of analogical inference task (Mikolov's dataset) by calculating the limited number of dimensions in word representation vectors. Success rates of DSI-decorr were not significantly different from DSI-sparse ($P>0.05$, 2-sample t-test) but higher than all other methods ($P<0.01$, 2-sample t-test for DSI (non-neg. OFF) and 1-sample t-tests for other word embedding methods) in the condition of 2, 4, 6, 8, and 10 dimensions, except DSI-decorr VS DSI-sparse in 2 dimensions ($P<0.05$) and DSI-decorr VS DSI (non-neg. OFF) in 10 dimensions ($P>0.05$). **$P<0.01$; n.s., not significant. All statistical tests were two-sided t-tests and significance thresholds were modified by Bonferroni correction.

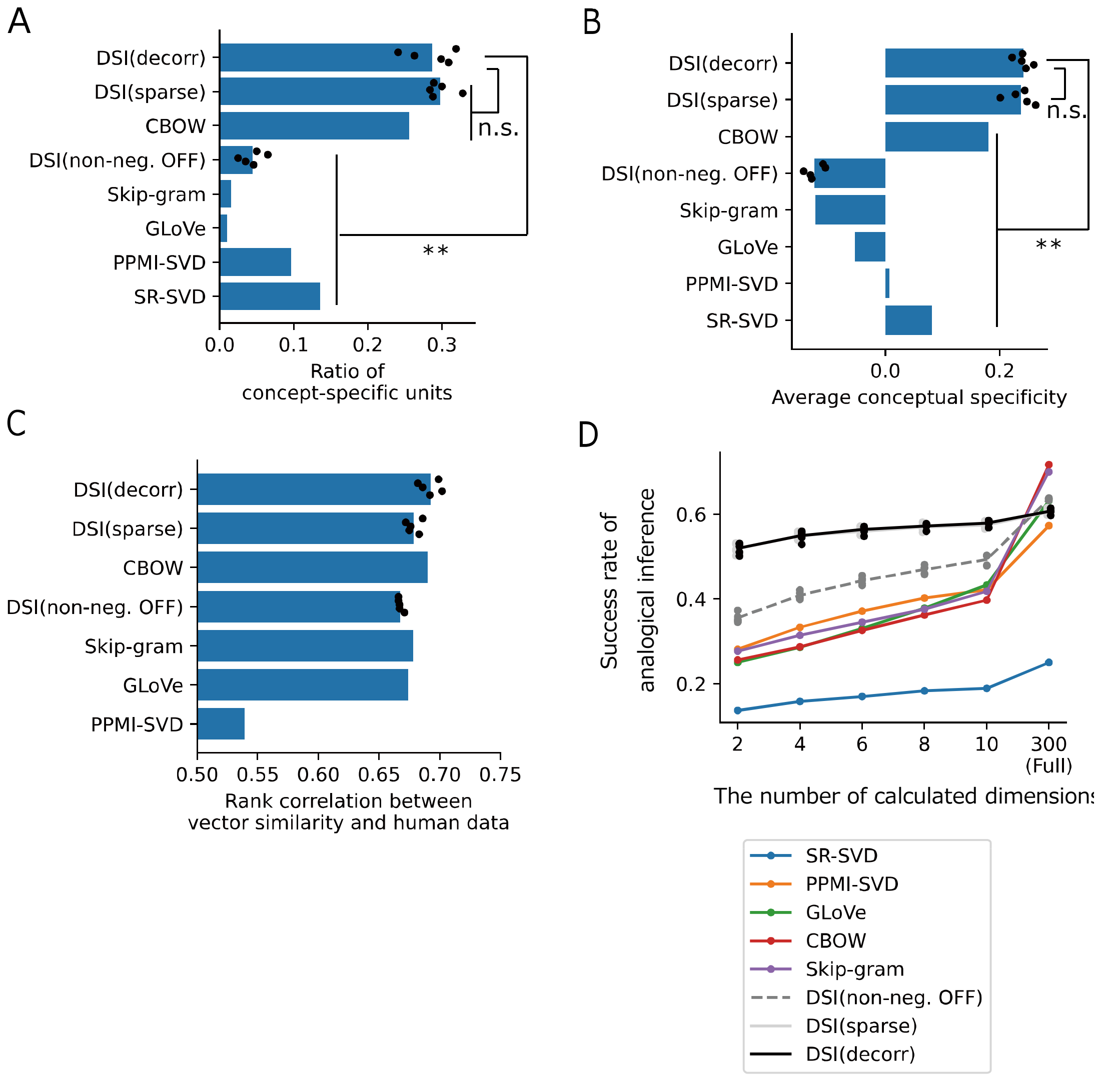

Fig. S7. Evaluation of word representations obtained by DSI and other word embedding methods (D=200). For DSI, dots indicate 5 trisals with different random seeds (different initial values for learning); bars and lines indicate means of those 5 simulations. (A) Ratio of concept-specific units. (B) Average conceptual specificity. (C) The rank correlation of word similarity evaluated by word representation vectors (cosine similarity) and humans (WS353 dataset). (D) Success rates of analogical inference task (Mikolov's dataset) by calculating the limited number of dimensions in word representation vectors. Success rates of DSI-decorr were not significantly different from DSI-sparse (P>0.05, 2-sample t-test) but higher than all other methods (P<0.01, 2-sample t-test for DSI (non-neg. OFF) and 1-sample t-tests for other word embedding methods) in the condition of 2, 4, 6, 8, and 10 dimensions. **P<0.01; n.s., not significant. All statistical tests were two-sided t-tests and significance thresholds were modified by Bonferroni correction.

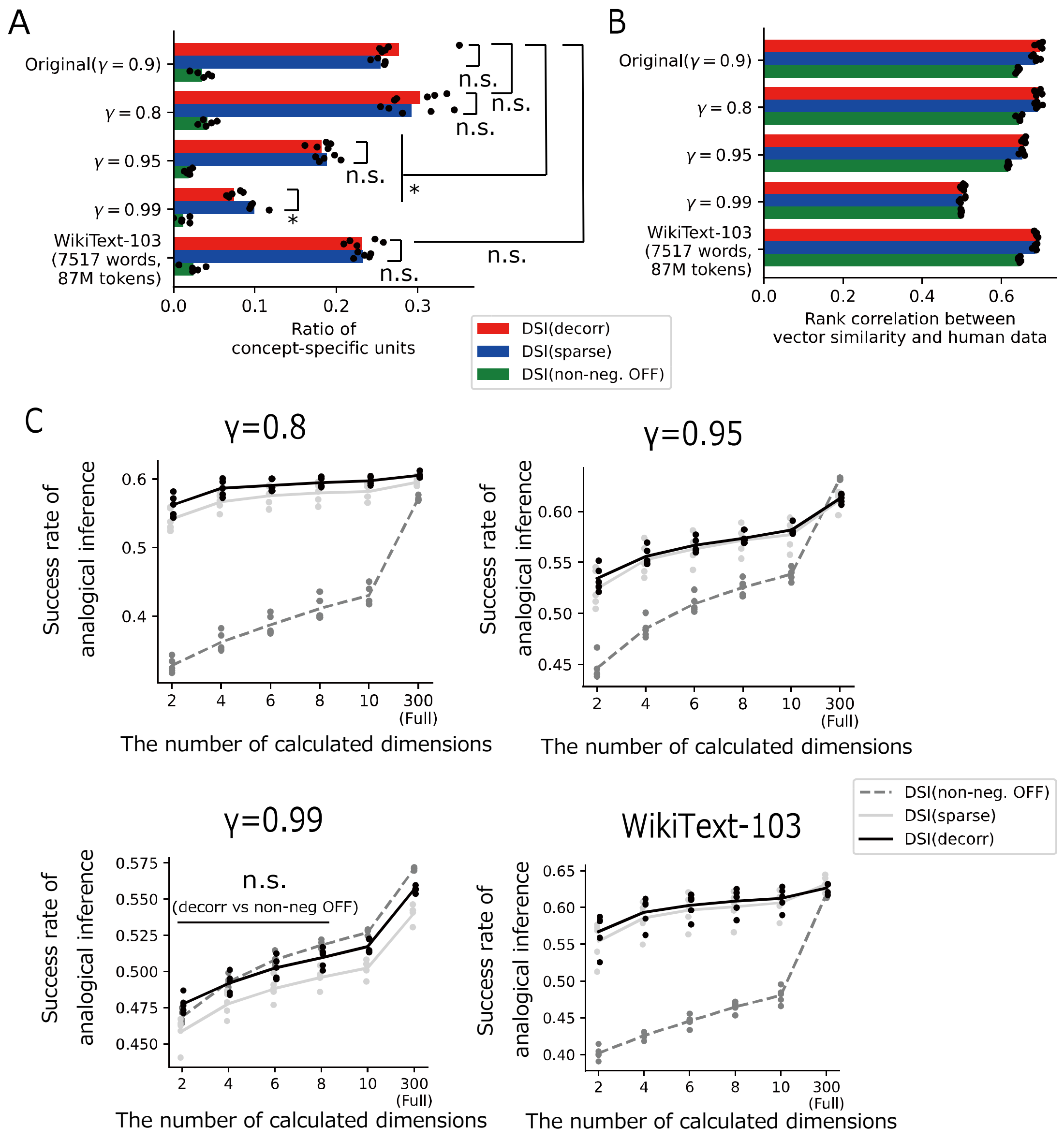

Fig. S8. Evaluation of DSI word representations obtained with various settings of γ ($\gamma = 0.8, 0.95, 0.99$) and a different dataset (WikiText-103) for learning. Other setting was same as the original one. Dots indicate 5 trials with different random seeds (different initial values for learning); bars and lines indicate means of those 5 simulations. (A) Ratio of concept-specific units. Statistical tests between different settings were performed for DSI-decorr. In each setting, DSI-decorr and DSI-sparse were compared. (B) The rank correlation of word similarity evaluated by word representation vectors (cosine similarity) and humans (WS353 dataset). (C) Success rates of analogical inference task (Mikolov's dataset) by calculating the limited number of dimensions in word representation vectors. In settings with $\gamma = 0.8, 0.95$ and WikiText-103, success rates of DSI-decorr were not significantly different from DSI-sparse (P>0.05, 2-sample t-test) but higher than all other methods (P<0.01, 2-sample t-test) in the condition of 2, 4, 6, 8, and 10 dimensions. In settings with $\gamma = 0.99$, success rates of DSI-decorr were not significantly

different from DSI (non-neg. OFF) ($P>0.05$, 2-sample t-test). *$P<0.05$; n.s., not significant. All statistical tests were two-sided t-tests and significance thresholds were modified by Bonferroni correction.

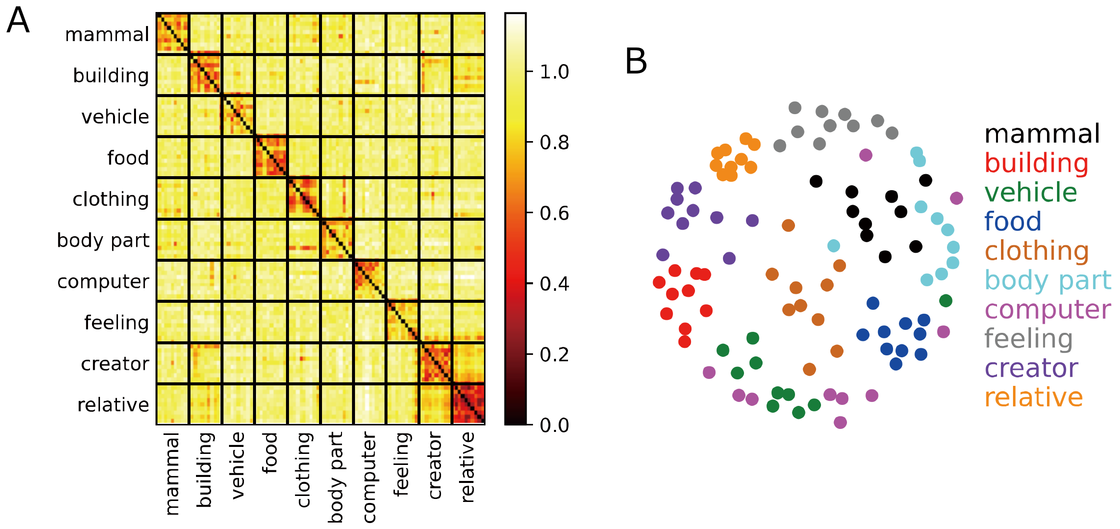

Fig. S9. DSI representations capture the semantic structure of words at a population level (DSI-sparse). (A) Dissimilarity matrix between DSI representation vectors for 100 words in 10 semantic categories. We selected 10 words in each category. We used same dissimilarity metric with Reber et al. (2019) (1 - Pearson's correlation coefficient). (B) Visualization of the representational structure of DSI using MDS based on the dissimilarity matrix. Each color corresponds to a semantic category.

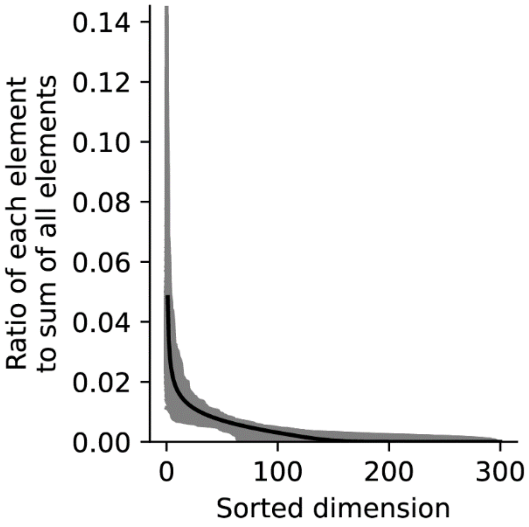

Fig. S10. Non-sparsity of word representations. Gray lines show ratio of each element to sum of all elements in each word representation vector, and the black line is the average of them. Elements were sorted in descending order.

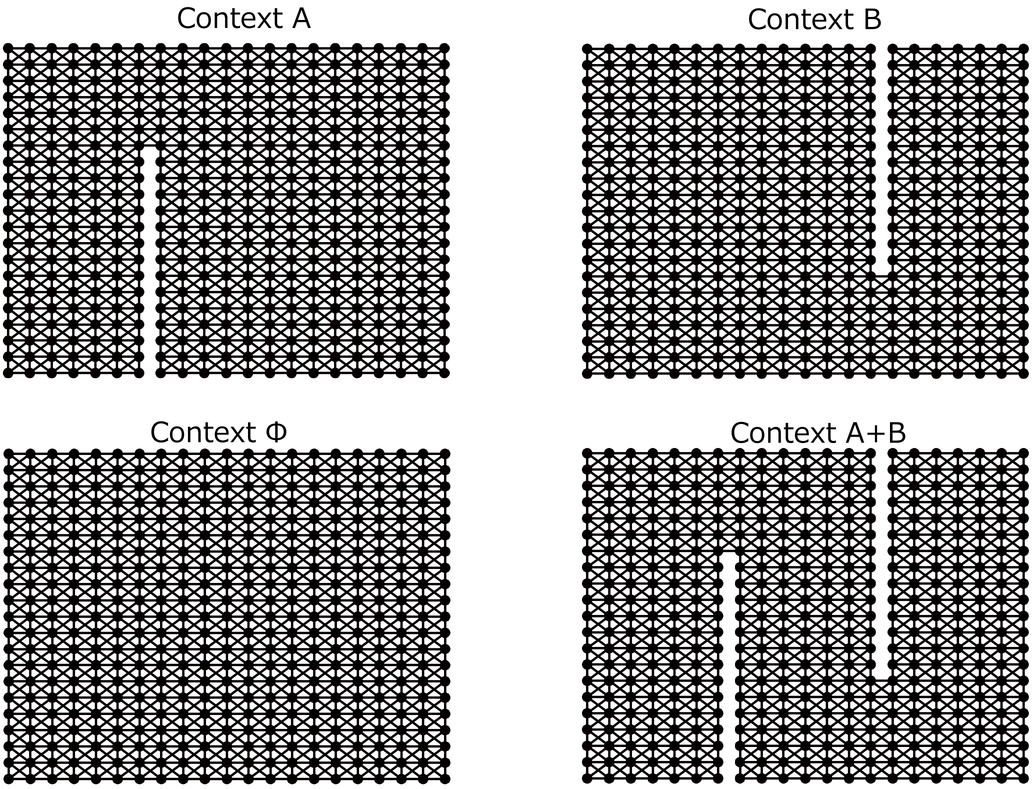

Fig. S11. Structures of state transition graphs used in inference task of spatial contexts.

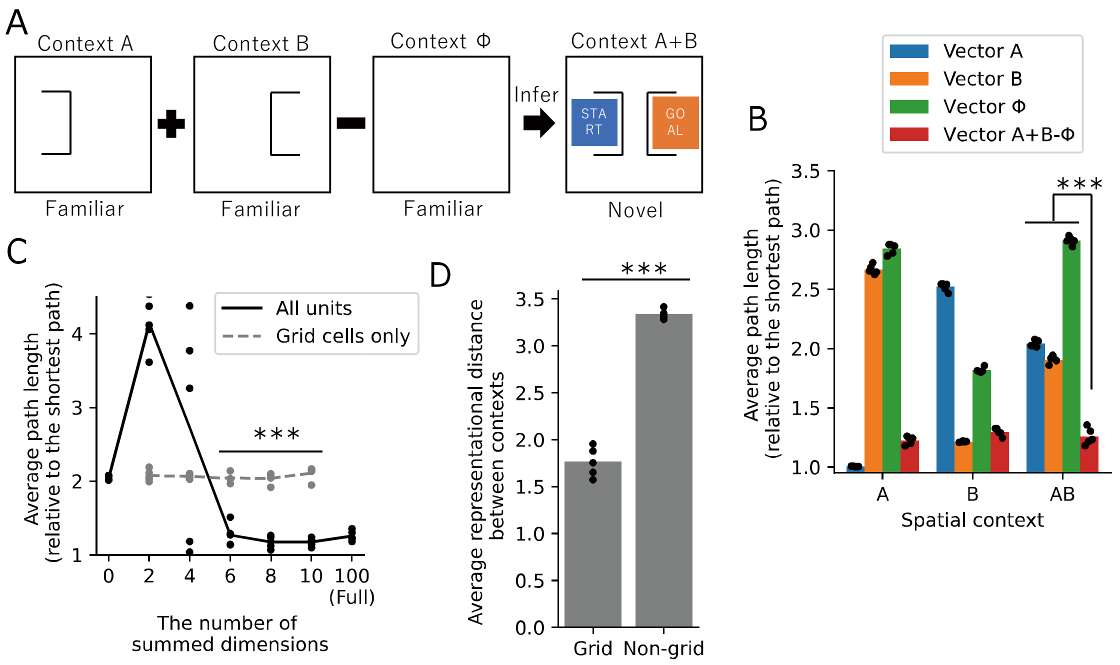

Fig. S12. Composite spatial representations enable navigation in a novel spatial context. (A) We constructed representation vectors for a novel context A+B by arithmetic composition of DSI representation vectors for three familiar contexts A, B, and Φ. The start and the goal in each navigation trial were randomly positioned in the colored area. (B) Average path lengths in 1,000 trials of spatial navigation under various settings of representation vectors and contexts. Note that we normalized a path length by the shortest path length between the start and the goal in each trial. Dots indicate 5 simulations with different random seeds (different initial values for learning and simulations); bars indicate means of those 5 simulations. (C) Average path lengths by the spatial navigation using the composite representation vectors in which we summed only the limited number of dimensions. Dots indicate 5 simulations with different random seeds (different initial values for learning and simulations); bars indicate means of those 5 simulations. 2-sample t-tests were performed between the condition in which we calculated only grid cells and the condition in which we calculated all units. (D) Average representational distances between the context B and Φ across of grid-like and non-grid-type units. Dots indicate 5 simulations with different random seeds (different initial values for learning and simulations); bars indicate means of those 5 simulations. ***P<0.001. All statistical tests were two-sided t-tests and significance thresholds were modified by Bonferroni correction. Details of statistical analyses are shown in Table S3.

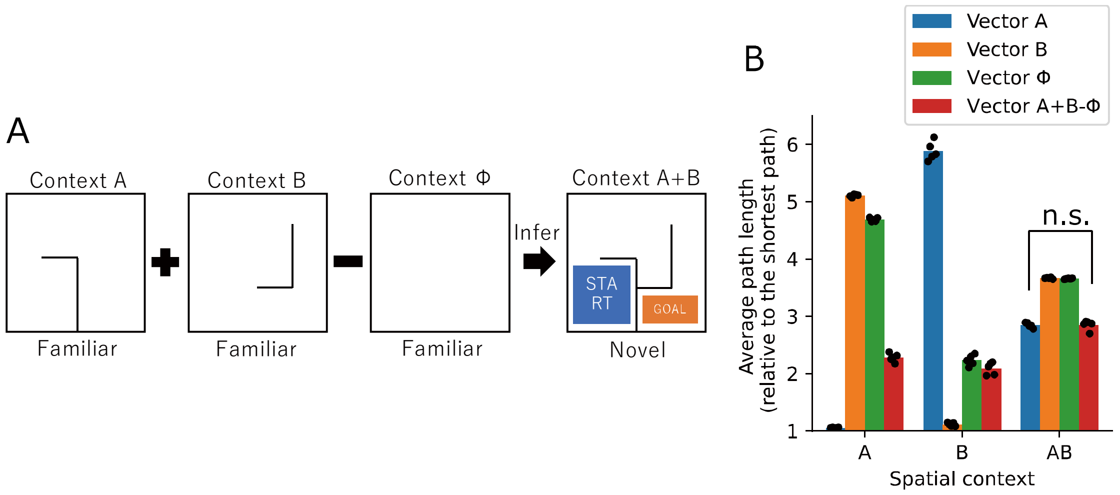

Fig. S13. An example in which composite spatial representations did not enable navigation in a novel spatial context. (A) We constructed representation vectors for a novel context A+B by arithmetic composition of DSI representation vectors for three familiar contexts A, B, and Φ. The start and the goal in each navigation trial were randomly positioned in the colored area. (B) Average path lengths in 1,000 trials of spatial navigation under various settings of representation vectors and contexts. Note that we normalized a path length by the shortest path length between the start and the goal in each trial. Dots indicate 5 simulations with different random seeds (different initial values for learning and simulations); bars indicate means of those 5 simulations. n.s., not significant. All statistical tests were two-sided t-tests and significance thresholds were modified by Bonferroni correction. Details of statistical analyses are shown in Table S3.

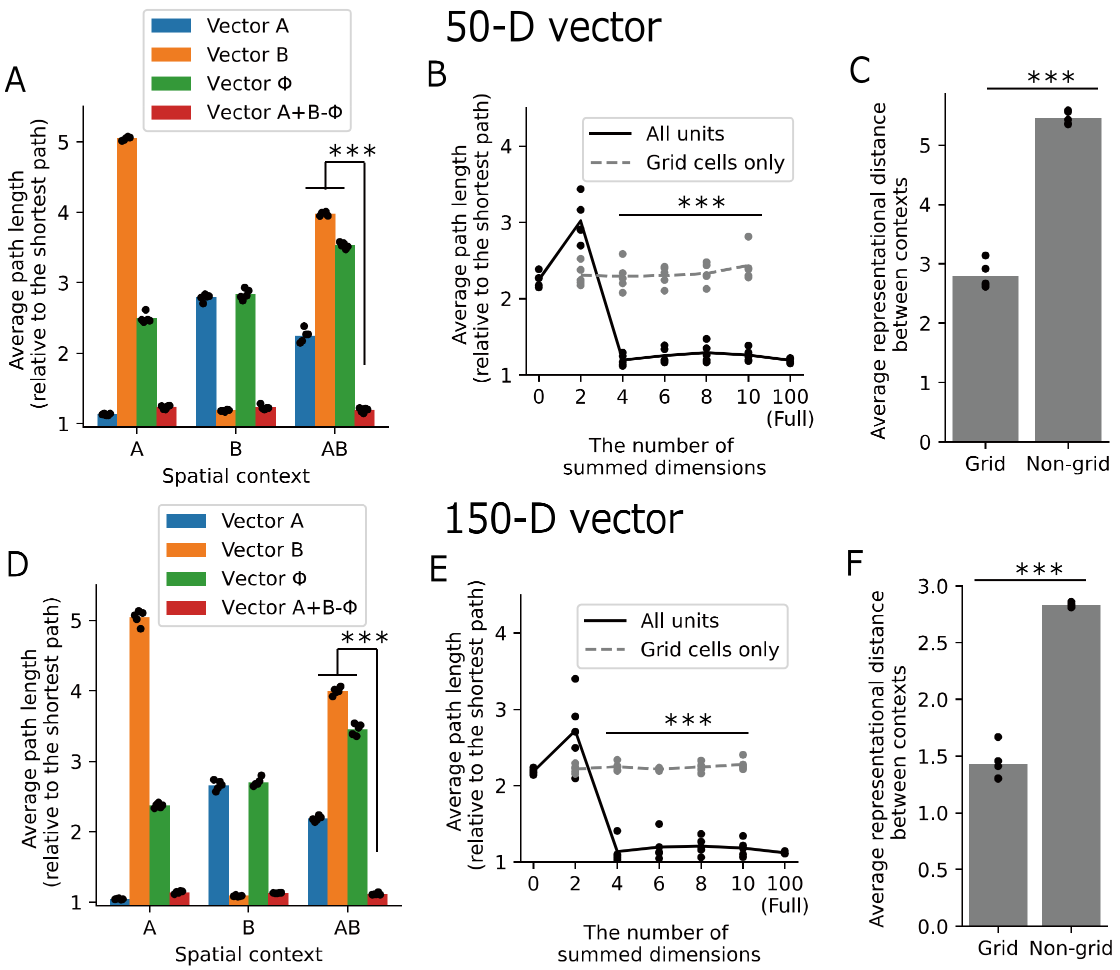

Fig. S14. The performance of analogical inference of spatial contexts by representation vectors with different dimensionality (D=50 and D=150). We used the spatial structure shown in Fig. 6 and Fig. S11. (A) Average path lengths in 1,000 trials of spatial navigation under various settings of representation vectors and contexts (D=50). Note that we normalized a path length by the shortest path length between the start and the goal in each trial. (B) Average path lengths by the spatial navigation using the composite representation vectors in which we summed only the limited number of dimensions (D=50). 2-sample t-tests were performed between the condition in which we calculated only grid cells and the condition in which we calculated all units. (D) Average representational distances between the context B and Φ of grid-like and non-grid-type units (D=50). (D, E, F) Same results obtained with D=150. In all figures, dots indicate 5 simulations with different random seeds (different initial values for learning and simulations); lines and bars indicate means of those 5 simulations. ***P<0.001. All statistical tests were two-sided t-tests and significance thresholds were modified by Bonferroni correction.

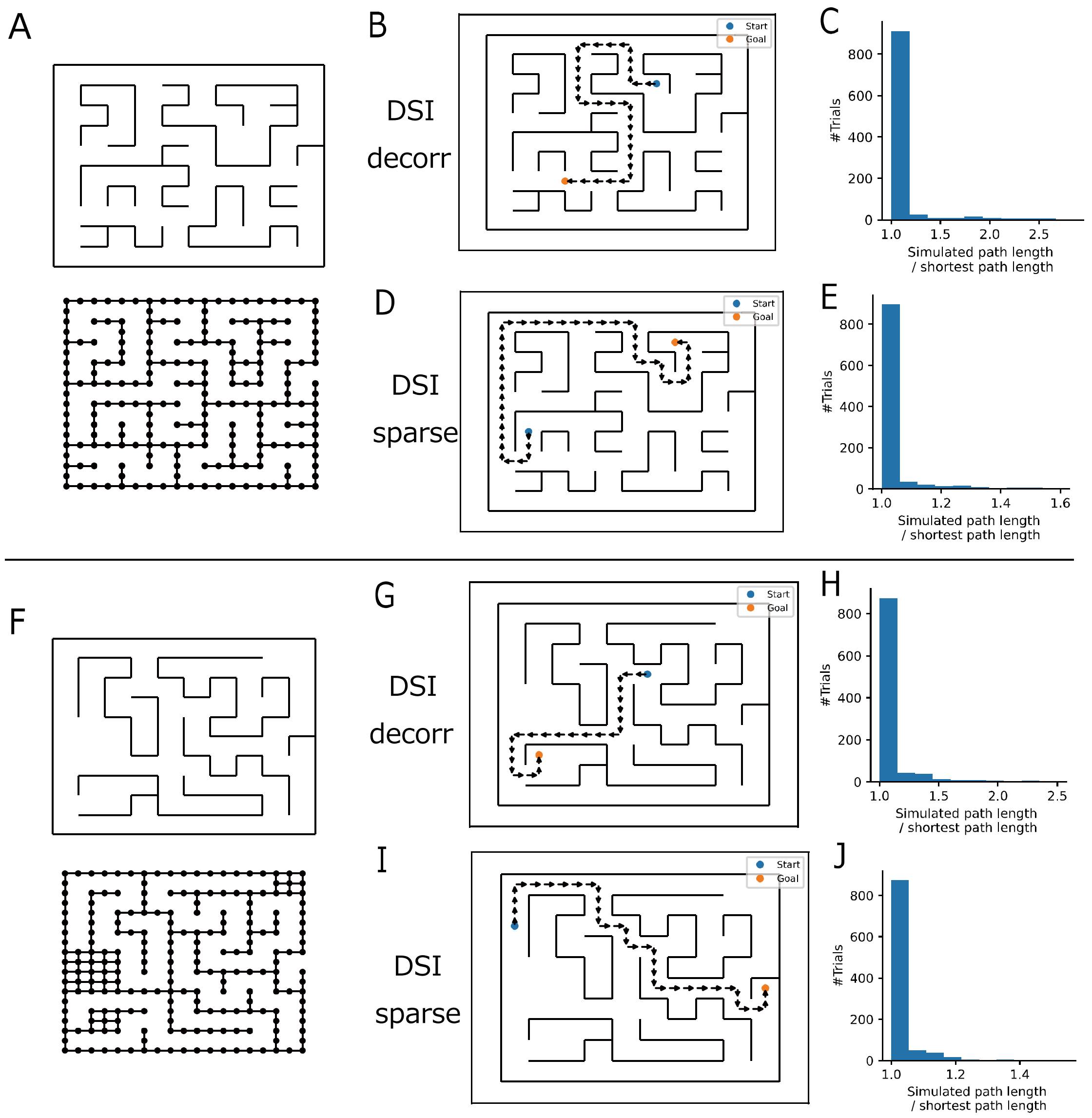

Fig. S15. Spatial navigation in complex mazes by DSI (A) The structure of the first maze (Upper) and the corresponding state transition graphs used for learning and navigation (Lower). (B, C) Results of navigation in the first maze by DSI-decorr. (B) An example trial in which the agent took the shortest path between the start and the goal. (C) A histogram of path lengths in 1,000 trials of spatial navigation in the first maze by DSI-decorr. Note that a start and a goal were randomly determined in each trial, and we normalized a simulated path length by the shortest path length between the start and the goal. (D, E) Results of navigation in the first maze by DSI-sparse. (F) The structure of the second maze (Upper) and the corresponding state transition graphs used for learning and navigation (Lower). (G, H) Results of navigation in the second maze by DSI-decorr. (I, J) Results of navigation in the second maze by DSI-sparse.

Table S1. Details of statistical analyses in Figure 2.

Comparison	Statistical test	Statistic	p-value
Figure 2B			
DSI (decorr) vs DSI (sparse)	Two-sided 2-sample t-test	$t(8)=1.2$	$p=0.27$
DSI (decorr) vs CBOW	Two-sided 1-sample t-test	$t(4)=1.2$	$p=0.91$
DSI (decorr) vs DSI (non-neg. OFF)	Two-sided 2-sample t-test	$t(8)=12.5$	$p=1.5\times10^{-6}$
DSI (decorr) vs Skip-gram	Two-sided 1-sample t-test	$t(4)=12.3$	$p=2.5\times10^{-4}$
DSI (decorr) vs GLoVe	Two-sided 1-sample t-test	$t(4)=13.8$	$p=1.6\times10^{-4}$
DSI (decorr) vs PPMI-SVD	Two-sided 1-sample t-test	$t(4)=8.4$	$p=0.0011$
DSI (decorr) vs SR-SVD	Two-sided 1-sample t-test	$t(4)=8.0$	$p=0.0013$
DSI (decorr) vs BERT	Two-sided 1-sample t-test	$t(4)=11.1$	$p=3.8\times10^{-4}$
Figure 2C			
DSI (decorr) vs DSI (sparse)	Two-sided 2-sample t-test	$t(8)=1.4$	$p=0.21$
DSI (decorr) vs CBOW	Two-sided 1-sample t-test	$t(4)=0.9$	$p=0.41$
DSI (decorr) vs DSI (non-neg. OFF)	Two-sided 2-sample t-test	$t(8)=17.6$	$p=1.1\times10^{-7}$
DSI (decorr) vs Skip-gram	Two-sided 1-sample t-test	$t(4)=17.5$	$p=6.2\times10^{-5}$
DSI (decorr) vs GLoVe	Two-sided 1-sample t-test	$t(4)=15.7$	$p=9.5\times10^{-5}$
DSI (decorr) vs PPMI-SVD	Two-sided 1-sample t-test	$t(4)=9.1$	$p=8.2\times10^{-4}$
DSI (decorr) vs SR-SVD	Two-sided 1-sample t-test	$t(4)=8.3$	$p=0.0011$
DSI (decorr) vs BERT	Two-sided 1-sample t-test	$t(4)=10.7$	$p=4.3\times10^{-4}$

Table S2. Details of statistical analyses in Figure 4D.

Number of calculated dimensions	n=2	n=4	n=6	n=8	n=10
DSI (decorr) vs DSI (sparse), Two-sided 2-sample t-test	$t(8)=1.9$, $p=0.10$	$t(8)=1.3$, $p=0.22$	$t(8)=1.6$, $p=0.14$	$t(8)=1.1$, $p=0.21$	$t(8)=1.2$, $p=0.28$
DSI (decorr) vs DSI (non-neg. OFF), Two-sided 2-sample t-test	$t(8)=22.9$, $p=1.4\times10^{-8}$	$t(8)=18.1$, $p=8.9\times10^{-8}$	$t(8)=17.4$, $p=1.2\times10^{-7}$	$t(8)=16.8$, $p=1.6\times10^{-7}$	$t(8)=12.3$, $p=1,8\times10^{-6}$
DSI (decorr) vs CBOW, Two-sided 1-sample t-test	$t(4)=45.2$, $p=1.4\times10^{-6}$	$t(4)=32.5$, $p=5.4\times10^{-6}$	$t(4)=34.1$, $p=4.4\times10^{-6}$	$t(4)=34.7$ $p=4.1\times10^{-6}$	$t(4)=28.8$, $p=8.7\times10^{-6}$
DSI (decorr) vs Skip-gram, Two-sided 1-sample t-test	$t(4)=40.1$, $p=2.3\times10^{-6}$	$t(4)=29.7$, $p=7.6\times10^{-6}$	$t(4)=32.5$, $p=5.3\times10^{-6}$	$t(4)=33.6$, $p=4.7\times10^{-6}$	$t(4)=28.5$, $p=9.0\times10^{-6}$
DSI (decorr) vs GLoVe, Two-sided 1-sample t-test	$t(4)=39.8$, $p=2.4\times10^{-6}$	$t(4)=30.7$, $p=6.7\times10^{-6}$	$t(4)=33.9$, $p=4.5\times10^{-6}$	$t(4)=37.5$, $p=3.0\times10^{-6}$	$t(4)=32.5$, $p=5.3\times10^{-6}$
DSI (decorr) vs PPMI-SVD, Two-sided 1-sample t-test	$t(4)=41.7$, $p=2.0\times10^{-6}$	$t(4)=28.6$, $p=8.9\times10^{-6}$	$t(4)=28.8$, $p=8.7\times10^{-6}$	$t(4)=30.1$, $p=7.2\times10^{-6}$	$t(4)=25.1$, $p=1.5\times10^{-4}$
DSI (decorr) vs SR-SVD, Two-sided 1-sample t-test	$t(4)=59.0$, $p=5.0\times10^{-7}$	$t(4)=46.3$, $p=1.3\times10^{-6}$	$t(4)=53.4$, $p=7.4\times10^{-7}$	$t(4)=61.7$, $p=4.1\times10^{-7}$	$t(4)=56.9$, $p=5.7\times10^{-7}$

Table S3. Details of statistical analyses in Figure 6, Figure S12 and S13. p=0 indicates a value smaller than the minimum of the floating-point number.

Comparison	Statistical test	Statistic	p-value
Figure 6C			
Vector A+B-Φ vs Vector A	Two-sided 2-sample t-test	t(8)=52.1	p=0
Vector A+B-Φ vs Vector B	Two-sided 2-sample t-test	t(8)=90.3	p=0
Vector A+B-Φ vs VectorΦ	Two-sided 2-sample t-test	t(8)=96.8	p=0
Figure 6E			
All units vs Grid cells only, n=2	Two-sided 2-sample t-test	t(8)=2.4	p=0.043
All units vs Grid cells only, n=4	Two-sided 2-sample t-test	t(8)=46.7	p=0
All units vs Grid cells only, n=6	Two-sided 2-sample t-test	t(8)=20.5	p=0
All units vs Grid cells only, n=8	Two-sided 2-sample t-test	t(8)=22.1	p=0
All units vs Grid cells only, n=10	Two-sided 2-sample t-test	t(8)=28.3	p=0
Figure 6H			
Grid cells vs Non-grid cells	Two-sided 2-sample t-test	t(8)=24.5	p=0
Figure S7B			
Vector A+B-Φ vs Vector A	Two-sided 2-sample t-test	t(8)=23.1	p=0
Vector A+B-Φ vs Vector B	Two-sided 2-sample t-test	t(8)=18.9	p=0
Vector A+B-Φ vs VectorΦ	Two-sided 2-sample t-test	t(8)=47.3	p=0
Figure S12C			
All units vs Grid cells only, n=2	Two-sided 2-sample t-test	t(8)=12.8	$p=1\times10^{-6}$
All units vs Grid cells only, n=4	Two-sided 2-sample t-test	t(8)=0.97	p=0.36
All units vs Grid cells only, n=6	Two-sided 2-sample t-test	t(8)=10.4	$p=6\times10^{-6}$
All units vs Grid cells only, n=8	Two-sided 2-sample t-test	t(8)=17.9	p=0
All units vs Grid cells only, n=10	Two-sided 2-sample t-test	t(8)=19.4	p=0
Figure S12D			
Grid cells vs Non-grid cells	Two-sided 2-sample t-test	t(8)=21.3	p=0
Figure S13B			
Vector A+B-Φ vs Vector A	Two-sided 2-sample t-test	t(8)=0.03	p=0.98

SI References

1. E. Todorov, Linearly-solvable Markov decision problems in *Advances in Neural Information Processing Systems 19*, (2006), pp. 1369–1376.
2. E. Todorov, Efficient computation of optimal actions. *Proc Natl Acad Sci U S A* **106**, 11478–11483 (2009).
3. P. Piray, N. D. Daw, Linear reinforcement learning in planning, grid fields, and cognitive control. *Nat Commun* **12** (2021).
4. P. Dayan, Improving Generalization for Temporal Difference Learning: The Successor Representation. *Neural Comput* **5**, 613–624 (1993).
5. K. L. Stachenfeld, M. M. Botvinick, S. J. Gershman, The hippocampus as a predictive map. *Nat Neurosci* **20**, 1643–1653 (2017).
6. Y. E. Nesterov, A method of solving a convex programming problem with convergence rate O(1/k^2). *Dokl Akad Nauk* **269**, 543–547 (1983).
7. X. Zuo, mazelab: A customizable framework to create maze and gridworld environments. *https://github.com/zuoxingdong/mazelab* (2018).
8. F. Sargolini, *et al.*, Conjunctive Representation of Position, Direction, and Velocity in Entorhinal Cortex. *Science (1979)* **312**, 758–762 (2006).
9. R. F. Langston, *et al.*, Development of the Spatial Representation System in the Rat. *Science (1979)* **328**, 1576–1580 (2010).
10. A. Banino, *et al.*, Vector-based navigation using grid-like representations in artificial agents. *Nature* **557**, 429–433 (2018).
11. C. Barry, N. Burgess, To be a Grid Cell: Shuffling procedures for determining "Gridness." *bioRxiv* (2017). https://doi.org/10.1101/230250.
12. S. Merity, C. Xiong, J. Bradbury, R. Socher, Pointer Sentinel Mixture Models in *International Conference on Learning Representations*, (2017).
13. T. Mikolov, K. Chen, G. Corrado, J. Dean, Distributed Representations of Words and Phrases and their Compositionality. *ArXiv* (2013).
14. T. Mikolov, K. Chen, G. Corrado, J. Dean, Efficient Estimation of Word Representations in Vector Space. *ArXiv* (2013).
15. J. Pennington, R. Socher, C. Manning, Glove: Global Vectors for Word Representation in *Proceedings of the 2014 Conference on Empirical Methods in Natural Language Processing (EMNLP)*, (Association for Computational Linguistics, 2014), pp. 1532–1543.

16. O. Levy, Y. Goldberg, Neural Word Embedding as Implicit Matrix Factorization in *Advances in Neural Information Processing Systems 7*, (2014), pp. 2177–2185.
17. J. Devlin, M.-W. Chang, K. Lee, K. Toutanova, BERT: Pre-training of Deep Bidirectional Transformers for Language Understanding. *ArXiv* (2018).
18. T. Wolf, *et al.*, Transformers: State-of-the-Art Natural Language Processing in *Proceedings of the 2020 Conference on Empirical Methods in Natural Language Processing: System Demonstrations*, (2020), pp. 38–45.
19. Princeton University, About Wordnet. *https://wordnet.princeton.edu/* (2010).
20. E. Oja, M. Plumbley, Blind Separation of Positive Sources by Globally Convergent Gradient Search. *Neural Comput* **16**, 1811–1825 (2004).
21. A. Hyvärinen, E. Oja, Independent component analysis: algorithms and applications. *Neural Networks* **13**, 411–430 (2000).
22. J. C. R. Whittington, W. Dorrell, S. Ganguli, S. Timothy, E. J. Behrens, Disentanglement with Biological Constraints: A Theory of Functional Cell Types in *The Eleventh International Conference on Learning Representations*, (2022).
23. I. Higgins, *et al.*, Unsupervised deep learning identifies semantic disentanglement in single inferotemporal face patch neurons. *Nat Commun* **12** (2021).
24. T. P. Reber, *et al.*, Representation of abstract semantic knowledge in populations of human single neurons in the medial temporal lobe. *PLoS Biol* **17** (2019).
25. R. Q. Quiroga, Concept cells: the building blocks of declarative memory functions. *Nat Rev Neurosci* **13**, 587–597 (2012).
26. Ø. A. Høydal, E. R. Skytøen, S. O. Andersson, M. B. Moser, E. I. Moser, Object-vector coding in the medial entorhinal cortex. *Nature* **568**, 400–404 (2019).
27. K. C. Bittner, A. D. Milstein, C. Grienberger, S. Romani, J. C. Magee, Behavioral time scale synaptic plasticity underlies CA1 place fields. *Science (1979)* **357**, 1033–1036 (2017).
28. Y. Dordek, D. Soudry, R. Meir, D. Derdikman, Extracting grid cell characteristics from place cell inputs using non-negative principal component analysis. (2016). https://doi.org/10.7554/eLife.10094.001.
29. B. Sorscher, G. C. Mel, S. Ganguli, S. A. Ocko, A unified theory for the origin of grid cells through the lens of pattern formation in *Advances in Neural Information Processing Systems*, (2019), pp. 10003–10013.
30. B. Sorscher, G. C. Mel, S. A. Ocko, L. M. Giocomo, S. Ganguli, A unified theory for the computational and mechanistic origins of grid cells. *Neuron* **111**, 121-137.e13 (2023).

31. B. Murphy, P. P. Talukdar, T. Mitchell, Learning Effective and Interpretable Semantic Models using Non-Negative Sparse Embedding in *Proceedings of COLING 2012*, (2012), pp. 1933–1950.
32. L. Z. Fan, *et al.*, All-optical physiology resolves a synaptic basis for behavioral timescale plasticity. *Cell* **186**, 543-559.e19 (2023).
33. M. Brysbaert, How many words do we read per minute? A review and meta-analysis of reading rate. *J Mem Lang* **109** (2019).
34. B. A. Strange, M. P. Witter, E. S. Lein, E. I. Moser, Functional organization of the hippocampal longitudinal axis. *Nat Rev Neurosci* **15**, 655–669 (2014).
35. K. B. Kjelstrup, *et al.*, Finite Scale of Spatial Representation in the Hippocampus. *Science (1979)* **321**, 140–143 (2008).
36. I. K. Brunec, *et al.*, Multiple Scales of Representation along the Hippocampal Anteroposterior Axis in Humans. *Current Biology* **28**, 2129-2135.e6 (2018).
37. J. Robin, M. Moscovitch, Details, gist and schema: hippocampal–neocortical interactions underlying recent and remote episodic and spatial memory. *Curr Opin Behav Sci* **17**, 114–123 (2017).
38. I. Momennejad, Learning Structures: Predictive Representations, Replay, and Generalization. *Curr Opin Behav Sci* **32**, 155–166 (2020).
39. O. Baumann, J. B. Mattingley, Dissociable representations of environmental size and complexity in the human hippocampus. *Journal of Neuroscience* **33**, 10526–10533 (2013).
40. M. Contreras, T. Pelc, M. Llofriu, A. Weitzenfeld, J. M. Fellous, The ventral hippocampus is involved in multi-goal obstacle-rich spatial navigation. *Hippocampus* **28**, 853–866 (2018).
41. J. Liu, *et al.*, Multi-scale goal distance representations in human hippocampus during virtual spatial navigation. *Current Biology* **33**, 2024-2033.e3 (2023).
42. A. Montagrin, *et al.*, The hippocampus dissociates present from past and future goals. *Nat Commun* **15** (2024).
43. M. M. Botvinick, Hierarchical reinforcement learning and decision making. *Curr Opin Neurobiol* **22**, 956–962 (2012).
44. M. M. Botvinick, Y. Niv, A. C. Barto, Hierarchically organized behavior and its neural foundations: A reinforcement learning perspective. *Cognition* **113**, 262–280 (2009).
45. A. K. Lee, M. A. Wilson, Memory of Sequential Experience in the Hippocampus during Slow Wave Sleep. *Neuron* **36**, 1183–1194 (2002).

46. M. A. L. Ralph, E. Jefferies, K. Patterson, T. T. Rogers, The neural and computational bases of semantic cognition. *Nat Rev Neurosci* **18**, 42–55 (2017).
47. A. M. Saxe, J. L. McClelland, S. Ganguli, A mathematical theory of semantic development in deep neural networks. *Proc Natl Acad Sci U S A* **166**, 11537–11546 (2019).
48. T. T. Rogers, J. L. McClelland, Précis of Semantic Cognition: A Parallel Distributed Processing Approach. *Behavioral and Brain Sciences* **31**, 689–714 (2008).
49. T. M. Mitchell, *et al.*, Predicting Human Brain Activity Associated with the Meanings of Nouns. *Science (1979)* **320**, 1191–1195 (2008).
50. K. Patterson, P. J. Nestor, T. T. Rogers, Where do you know what you know? The representation of semantic knowledge in the human brain. *Nat Rev Neurosci* **8**, 976–987 (2007).
51. S. Nishida, S. Nishimoto, Decoding naturalistic experiences from human brain activity via distributed representations of words. *Neuroimage* **180**, 232–242 (2018).
52. A. O. Constantinescu, J. X. O'reilly, T. E. J. Behrens, Organizing conceptual knowledge in humans with a gridlike code. *Science (1979)* **352**, 1464–1468 (2016).
53. X. Bao, *et al.*, Grid-like Neural Representations Support Olfactory Navigation of a Two-Dimensional Odor Space. *Neuron* **102**, 1066-1075.e5 (2019).
54. S. A. Park, D. S. Miller, E. D. Boorman, Inferences on a multidimensional social hierarchy use a grid-like code. *Nat Neurosci* **24**, 1292–1301 (2021).
55. E. Z. Patai, H. J. Spiers, The Versatile Wayfinder: Prefrontal Contributions to Spatial Navigation. *Trends Cogn Sci* **25**, 520–533 (2021).
56. S. A. Bunge, C. Wendelken, D. Badre, A. D. Wagner, Analogical reasoning and prefrontal cortex: Evidence for separable retrieval and integration mechanisms. *Cerebral Cortex* **15**, 239–249 (2005).
57. B. L. McNaughton, F. P. Battaglia, O. Jensen, E. I. Moser, M. B. Moser, Path integration and the neural basis of the "cognitive map." *Nat Rev Neurosci* **7**, 663–678 (2006).
58. M. Gil, *et al.*, Impaired path integration in mice with disrupted grid cell firing. *Nat Neurosci* **21**, 81–91 (2018).
59. Y. Shrager, C. B. Kirwan, L. R. Squire, Neural basis of the cognitive map: Path integration does not require hippocampus or entorhinal cortex. *Proceedings of the National Academy of Sciences* **105**, 12034–12038 (2008).
60. C. Parron, E. Save, Evidence for entorhinal and parietal cortices involvement in path integration in the rat. *Exp Brain Res* **159**, 349–359 (2004).

61. A. M. P. Miller, L. C. Vedder, L. M. Law, D. M. Smith, Cues, context, and long-term memory: the role of the retrosplenial cortex in spatial cognition. *Front Hum Neurosci* **8** (2014).
